# Long non-coding RNAs identify a subset of luminal muscle-invasive bladder cancer patients with favorable prognosis

**DOI:** 10.1186/s13073-019-0669-z

**Published:** 2019-10-17

**Authors:** Joep J. de Jong, Yang Liu, A. Gordon Robertson, Roland Seiler, Clarice S. Groeneveld, Michiel S. van der Heijden, Jonathan L. Wright, James Douglas, Marc Dall’Era, Simon J. Crabb, Bas W. G. van Rhijn, Kim E. M. van Kessel, Elai Davicioni, Mauro A. A. Castro, Yair Lotan, Ellen C. Zwarthoff, Peter C. Black, Joost L. Boormans, Ewan A. Gibb

**Affiliations:** 1 Department of Urology, Erasmus MC Cancer Institute, Rotterdam, The Netherlands; 2 Decipher Biosciences, Inc, Vancouver, British Columbia Canada; 3 Canada’s Michael Smith Genome Sciences Center, BC Cancer Agency, Vancouver, British Columbia Canada; 4 Department of Urology, University of Bern, Bern, Switzerland; 5 Bioinformatics and Systems Biology Laboratory, Federal University of Paraná, Polytechnic Center, Curitiba, Brazil; 6 Department of Medical Oncology, Netherlands Cancer Institute, Amsterdam, The Netherlands; 7 Department of Urology, University of Washington School of Medicine, Seattle, WA USA; 8 Department of Urology, University Hospital of Southampton, Hampshire, UK; 9 UC Davis Comprehensive Cancer Center, Sacramento, CA USA; 10 Department of Medical Oncology, University Hospital of Southampton, Hampshire, UK; 11 Department of Surgical Oncology (Urology), Netherlands Cancer Institute – Antoni van Leeuwenhoek Hospital, Amsterdam, The Netherlands; 12 Department of Urology, UT Southwestern Medical Center, Dallas, TX USA; 13 Department of Pathology, Erasmus MC University Medical Center Rotterdam, Rotterdam, The Netherlands; 14 Department of Urologic Sciences, University of British Columbia, Vancouver, British Columbia Canada

**Keywords:** Gene expression analysis, Long non-coding RNA, Molecular subtypes, Muscle-invasive bladder cancer

## Abstract

**Background:**

Muscle-invasive bladder cancer (MIBC) is a heterogeneous disease, and gene expression profiling has identified several molecular subtypes with distinct biological and clinicopathological characteristics. While MIBC subtyping has primarily been based on messenger RNA (mRNA), long non-coding RNAs (lncRNAs) may provide additional resolution.

**Methods:**

LncRNA expression was quantified from microarray data of a MIBC cohort treated with neoadjuvant chemotherapy (NAC) and radical cystectomy (RC) (*n* = 223). Unsupervised consensus clustering of highly variant lncRNAs identified a four-cluster solution, which was characterized using a panel of MIBC biomarkers, regulon activity profiles, gene signatures, and survival analysis. The four-cluster solution was confirmed in The Cancer Genome Atlas (TCGA) cohort (*n* = 405). A single-sample genomic classifier (GC) was trained using ridge-penalized logistic regression and validated in two independent cohorts (*n* = 255 and *n* = 94).

**Results:**

NAC and TCGA cohorts both contained an lncRNA cluster (LC3) with favorable prognosis that was enriched with tumors of the luminal-papillary (LP) subtype. In both cohorts, patients with LP tumors in LC3 (LPL-C3) were younger and had organ-confined, node-negative disease. The LPL-C3 tumors had enhanced FGFR3, SHH, and wild-type p53 pathway activity. In the TCGA cohort, LPL-C3 tumors were enriched for *FGFR3* mutations and depleted for *TP53* and *RB1* mutations. A GC trained to identify these LPL-C3 patients showed robust performance in two validation cohorts.

**Conclusions:**

Using lncRNA expression profiles, we identified a biologically distinct subgroup of luminal-papillary MIBC with a favorable prognosis. These data suggest that lncRNAs provide additional information for higher-resolution subtyping, potentially improving precision patient management.

**Electronic supplementary material:**

The online version of this article (10.1186/s13073-019-0669-z) contains supplementary material, which is available to authorized users.

## Background

Bladder cancer has a global annual incidence of 430,000 patients, making it the fourth and tenth most common malignancy in men and women, respectively [1]. Approximately 25% of patients present with muscle-invasive bladder cancer (MIBC). The recommended treatment option for MIBC is neoadjuvant cisplatin-based chemotherapy (NAC) followed by pelvic lymph node dissection and radical cystectomy (RC) [2, 3]. Despite this aggressive treatment regimen, the 5-year overall survival (OS) is only approximately 55% from the time of surgery.

In recent years, gene expression profiling has revealed that MIBC is a heterogeneous disease; like breast cancer, it can be stratified into different molecular subtypes [4–7]. At the highest level, there is a division into basal and luminal subtypes, with different models providing additional subdivisions [8, 9]. Stratifying MIBC by molecular subtype has potential clinical value in terms of predicting both outcome and response to treatment, such as NAC or immunotherapy [10–12].

While most MIBC studies to date have exclusively used messenger RNA (mRNA) expression to differentiate molecular subtypes, the mammalian transcriptome is comprised of a diverse range of coding (mRNA) and non-coding RNAs. Long non-coding RNAs (lncRNAs) are mRNA-like transcripts that range in length from 200 nucleotides to over 100 kilobases and lack open reading frames [13]. They represent a significant fraction of the transcriptome, and, while it is unclear how many lncRNAs have biological function, their expression patterns can be specific to a particular biological or disease state [14, 15]. In the TCGA study, the lncRNA transcriptome divided the luminal-papillary subtype into two groups with distinct prognosis [12]. These findings suggest that lncRNA expression may offer additional resolution of molecular subtypes, potentially revealing additional prognostic information not captured by mRNA profiling.

In the present study, we aimed to expand these initial TCGA findings, further exploring the utility of lncRNA expression profiling for finer-grained molecular subtyping of MIBC.

## Methods

### Patient populations and expression data

For the present study, we analyzed four MIBC patient cohorts (Table 1). (1) NAC cohort: We compiled a cohort of 223 MIBC patients from seven institutions who had received neoadjuvant/induction chemotherapy followed by radical cystectomy (RC) for cT2-4aN0-3M0 urothelial carcinoma of the bladder [11]. Whole transcriptome profiling had previously been performed on formalin-fixed, paraffin-embedded (FFPE), pre-treatment tissue samples from transurethral bladder tumor resection (TURBT) in a Clinical Laboratory Improvement Amendments (CLIA)-certified laboratory (Decipher Biosciences, Inc., San Diego, CA) [16]. (2) TCGA cohort: RNA-seq data of 405 MIBC patients treated with RC in the absence of NAC was publicly available and previously analyzed by The Cancer Genome Atlas (TCGA) Research Network [12]. (3) PCC cohort: A prospective commercial cohort (PCC) consisting of the de-identified and anonymized gene expression profiles of 255 MIBC patients from the clinical use of the Decipher Bladder TURBT test that were available in the Decipher GRID registry (NCT02609269). Pathological staging and clinical outcome data were not available for this cohort. (4) UTSW cohort: The UT Southwestern (UTSW) cohort consisting of 94 MIBC patients from the UT Southwestern Medical Center who underwent RC without neoadjuvant therapy [17]. In this cohort, whole transcriptome profiling was performed on RC tissue samples. The NAC, PCC, and UTSW cohorts were all profiled on the GeneChip Human Exon 1.0 ST Array (Thermo Fisher, Carlsbad, CA). The lymphocyte and normal bladder expression datasets were downloaded directly from the GTEx Portal (https://gtexportal.org/).

**Table 1:** Clinicopathological characteristics of all patient cohorts

GC	Training	Testing	Validation	
Cohort	NAC [11]	TCGA [12]	UTSW	PCC
*N*	223	405	94	255
Tissue	TURBT	RC	RC	TURBT
Expression data	Array	RNA-seq	Array	Array
Age, median [IQR]	62 (56–71)	69 (60–76)	70 (63–77)	*NA*
Gender (%)				
Female	69 (31%)	106 (26%)	16 (17%)	*NA*
Male	154 (69%)	299 (74%)	78 (83%)	*NA*
Clinical lymph node stage (%)				
cN0	140 (63%)	*NA*	94 (100%)	*NA*
cN1–3	83 (37%)	*NA*	0 (0%)	*NA*
cNx	0 (0%)	*NA*	0 (0%)	*NA*
Clinical tumor stage (%)				
cTis/Ta	0 (0%)	*NA*	4 (4%)	*NA*
cT1	0 (0%)	*NA*	10 (11%)	*NA*
cT2	90 (40%)	*NA*	66 (70%)	*NA*
cT3	90 (40%)	*NA*	9 (10%)	*NA*
cT4	43 (20%)	*NA*	4 (4%)	*NA*
cTx	0 (0%)	*NA*	1 (1%)	*NA*
Pathological tumor stage (%)				
ypT0/Tis/Ta/T1	103 (46%)	0 (0%)	1 (1%)	*NA*
ypT2	42 (19%)	122 (30%)	36 (38%)	*NA*
ypT3	50 (22%)	193 (48%)	42 (45%)	*NA*
ypT4	24 (11%)	57 (14%)	15 (16%)	*NA*
ypTx	4 (2%)	33 (8%)	0 (0%)	*NA*
Pathological lymph node stage (%)				
ypN0	138 (62%)	235 (58%)	62 (66%)	*NA*
ypN1–3	48 (21%)	129 (32%)	31 (33%)	*NA*
ypNx	37 (17%)	41 (10%)	1 (1%)	*NA*
FGFR3+ cases by GC (%)	36 (16%)	55 (14%)	10 (11%)	24 (11%)
of which *n* died during follow-up:	2	9	1	*NA*

### Unsupervised clustering using lncRNAs

For unsupervised clustering analysis (R package ConsensusClusterPlus), the normalized gene expression data for *n* = 223 samples (NAC cohort) was pre-processed by multi-analysis distance sampling (R package MADS) to identify highly variant lncRNA genes. We assessed unsupervised consensus clustering with sets of between 250 and 1500 variant lncRNAs. After critically evaluating outputs from ConsensusClusterPlus (tracking plots, delta plots, CDF plots), we judged that the 750 lncRNA four-cluster solution was the most appropriate and informative. The expression clustering analysis was done by a consensus partitioning around medoids (PAM) approach, using Pearson correlations, and 10,000 iterations with a 0.95 random fraction of lncRNAs in each iteration. We repeated this process with log-transformed, RNA-seq gene expression data (TCGA cohort) for *n* = 405 samples to see whether clustering of our *de novo* selected lncRNA genes would identify lncRNA clusters that were similar to those identified by the TCGA analysis [12]. We determined concordance of this cluster solution with the published lncRNA cluster solution using Cohen’s kappa statistic.

### Classification of tumors among molecular mRNA subtypes

We generated a classifier that was based on the published TCGA 2017 mRNA subtypes [12], to classify tumors from the NAC, PCC, and UTSW cohorts into basal/squamous, luminal, luminal-infiltrated, luminal-papillary, and neuronal mRNA subtypes. We introduced an additional category, “unknown,” to provide a bin for tumors that did not fit the aforementioned subtyping structure. Furthermore, we applied the recently released consensus molecular classification by The Bladder Cancer Molecular Taxonomy Group to classify tumors from all four cohorts into six consensus mRNA subtypes: basal/squamous, luminal-papillary, luminal non-specified, luminal unstable, stroma-rich, and neuroendocrine-like [18].

### Regulon analysis of lncRNA clusters

Regulon analysis involves calculations that transform a cohort’s gene expression data into a functional readout that can inform on biological state [19, 20]. An initial step reconstructs regulatory units, each of which consists of a regulator, i.e., a gene whose product induces and/or represses a set of target genes, which we call a “regulon.” A second step calculates the activity profile of a regulon across a cohort. As demonstrated for breast cancer [19], and in the TCGA MIBC study [12], subsequent steps may use activity profiles as a molecular covariate to segregate clinical subtypes. In the work reported here, regulon activity profiles for both FGFR3 and SHH segregated FGFR3 and TP53 mutations, and the LPL-C3 tumors.

We used R package RTN v2.7.1 to calculate a transcriptional regulatory network from RSEM RNA-seq data for the TCGA-BLCA discovery cohort, as in Robertson et al. [12]. We used a set of 26 regulators: the 23 from TCGA work (*AR*, *EGFR*, *ERBB2*, *ERBB3*, *ESR1*, *ESR2*, *FGFR1*, *FGFR3*, *FOXA1*, *FOXM1*, *GATA3*, *GATA6*, *HIF1A*, *KLF4*, *PGR*, *PPARG*, *RARA*, *RARB*, *RARG*, *RXRA*, *RXRB*, *STAT3*, and *TP63*), with *RB1*, *SHH*, and *TP53* added. For calculating regulon activity profiles across a cohort, we required a regulon to have at least 15 positive and 15 negative targets. We used regulon target genes from the discovery cohort to calculate regulon activities in the NAC validation cohort. For each regulon, we performed enrichment tests (Fisher’s exact tests) to identify whether lncRNA clusters were enriched with samples of high or low regulon activity. We used RTNsurvival v1.6.0 and TCGA-BLCA mutation data [12] to generate oncoprint-like diagrams that showed, for the TCGA cohort, how regulon activity segregated *TP53* and *FGFR3* mutations, and LPL-C3 and LPL-Other samples.

### Gene expression analysis

We created heatmaps and boxplots to visualize differences between tumors from lncRNA and mRNA subtypes, in the expression of individual genes, gene signatures [5], and hallmark gene sets (from the molecular signature database hallmark gene set collection, MSigDB [21]). Hedgehog signaling activity was quantified by a signature based on target genes (*SHH*, *BMP4*, *BMP5*, *ID1*, *ID2*, *ID3*, *ID4*) as mentioned by Shin et al. [22]. *FGFR3* signaling was assessed by a gene signature from Sjödahl et al. [5]. Sample purity was calculated by the ABSOLUTE and ESTIMATE algorithms for the TCGA and NAC cohorts, respectively [23, 24]. Median fold changes (FC) and *p* values (using two-sided Wilcoxon rank-sum tests) were calculated for differential gene expression analyses. To identify lncRNAs enriched in immune cells, we filtered the GTEx datasets for lncRNAs with at least five median transcripts per million (TPM) higher expression in lymphocytes compared to a normal bladder. The candidate list of lncRNAs was compared to the 750 lncRNAs used for consensus clustering to generate a candidate list of immune-associated lncRNAs used for the clustering. The Immune190 signature score calculations have been previously described [25].

### Statistical analyses

Statistical analyses were performed using R statistical software (R Foundation for Statistical Computing, Vienna, Austria). In the NAC and TCGA cohorts, patient and tumor characteristics were compared between subgroups by Fisher’s exact tests and two-sided Wilcoxon rank-sum tests. *p* values for boxplot figures were determined by comparing LPL-C3 with LPL-other tumors by Wilcoxon rank-sum tests. The primary endpoint for the survival analysis was overall survival (OS). OS was calculated as the date of the most recent TURBT (NAC and PCC cohorts) or RC (TCGA and UTSW cohorts) till date of death from any cause. Patients who were lost to follow-up were censored at the date of last contact. The Kaplan-Meier method was used to estimate the statistical significance of differences between survival curves for patients of different molecular subtypes, using the log-rank test. After checking the proportional hazard assumption based on Schoenfeld residuals, we used multivariate Cox proportional hazard models to demonstrate the relationship of the genomic classifier’s predicted subtype with OS, adjusting for clinical variables, including age, sex, and stage.

### Discovery and validation of a genomic classifier

The NAC cohort was used to train a genomic classifier (GC) to predict luminal-papillary MIBC patients that had favorable prognosis (OS), as identified by the lncRNA clustering (LPL-C3). To make the model applicable to several platforms, we selected genes that were present in both the Illumina HiSeq platform (TCGA cohort) and Affymetrix Human Exon 1.0 ST Array (NAC, PCC, and UTSW cohorts) as the initial gene list (25,942 genes). Using this gene list, the selection of genes for the GC was based on an overlap of gene sets that were created by differential gene expression analyses (median FC < − 0.06 or > 0.1, *p* < 0.001), in which we compared lncRNA clusters and mRNA subtypes. This resulted in a list of 69 candidate genes. The final gene set included 65 genes after removing highly abundant mitochondrial transcripts (seven genes) and adding three genes enriched in LPL-C3, determined from heatmaps generated in the study (*SHH*, *BMP5*, and *FGFR3*) (Additional file 1: Table S1). Next, we trained a 10-fold cross-validated, ridge-penalized logistic regression model (R package glmnet) consisting of 36 coefficients to predict LPL-C3 MIBC (Additional file 1: Table S1). This model was applied to RNA-seq data (TCGA) using quantile normalization. For the 65 genes, expression values from RNA-seq were normalized by quantile-quantile matching with the expression values in our training cohort (NAC) as implemented in R package preprocessCore. We used the R package OptimalCutpoints to select the optimal probability threshold (*Pt*), corresponding to the maximal specificity for identifying LPL-C3 MIBC patients in both NAC and TCGA cohorts. Finally, we selected a probability threshold (*Pt*) of 0.43, corresponding to a 98–68% specificity-sensitivity combination in the NAC cohort and a 96–55% specificity-sensitivity combination in TCGA cohort. After training and testing of the GC in NAC and TCGA cohorts, the classifier was locked for further independent external validation in the PCC and UTSW cohorts.

## Results

### LncRNA expression profiling subdivides the luminal-papillary mRNA subtype

**Fig. 1:**
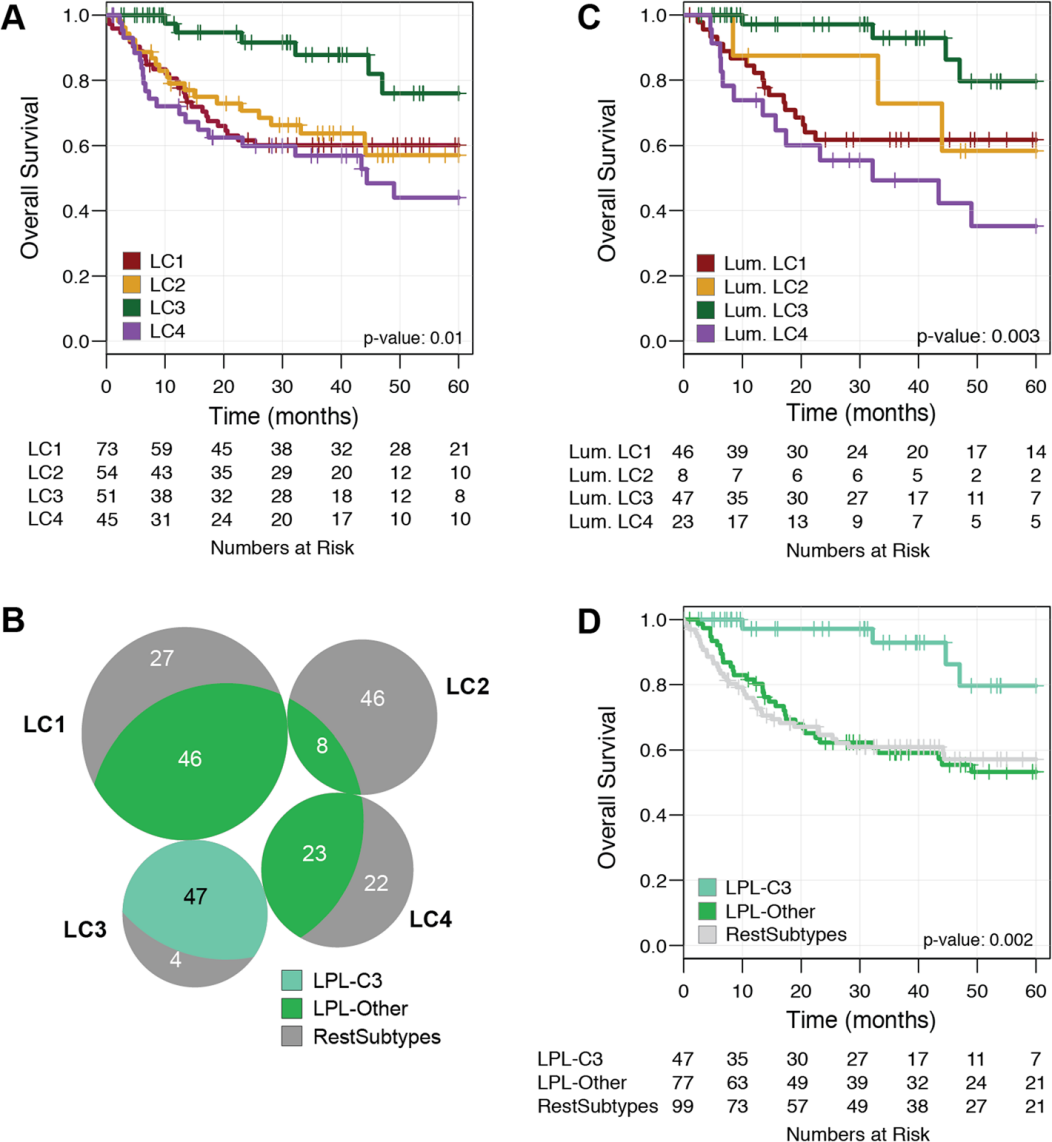
Survival analysis for the lncRNA-based clustering solution in the NAC cohort. **a** KM plot for lncRNA clusters (LC1–4), **b** intersection of the lncRNA clusters (LC1–4) with the luminal-papillary mRNA subtype, **c** KM plot for luminal-papillary mRNA subtype stratified by the lncRNA clusters, and **d** KM plot for lncRNA-split luminal-papillary tumors (LPL-C3, LPL-Other)

To explore the lncRNA expression landscape of MIBC, we downloaded a microarray-based cohort of 223 bladder cancer TURBT samples treated with NAC and RC (NAC cohort). Unsupervised consensus clustering of 750 of the most highly variant lncRNAs resulted in a robust four-cluster consensus solution (Additional file 2: Figure S1). Survival analysis of the lncRNA-based consensus clusters (LC1–4) revealed that LC3 had significantly better prognosis than clusters LC1, LC2, and LC4 (*p* = 0.01) (Fig. 1a).

To assign the tumors in the NAC cohort to TCGA 2017 mRNA subtypes (luminal-papillary, luminal, luminal-infiltrated, basal squamous and neuronal), we applied our single-sample classifier (Methods), which revealed that these tumors were enriched for basal/squamous (33%) and luminal-papillary (54%) subtypes (Additional file 2: Figure S2a). Survival analysis showed that patients with luminal-papillary tumors had better outcomes than the other subtypes (Additional file 2: Figure S2b).

Comparing our lncRNA four-cluster solution and the classifier assigned TCGA subtypes, we found LC2 was strongly enriched (72%, 39/54) for tumors of the basal/squamous subtype, whereas LC1, LC3, and LC4 contained only 23%, 4%, and 33% basal/squamous tumors, respectively (*p* < 0.001). Conversely, luminal-papillary tumors were enriched in LC3 (92%, 47/51) but were also present in LC1 (63%) and LC4 (51%) clusters (*p* < 0.001) (Fig. 1b). Considering only the luminal-papillary subtype (*n* = 124), we found patients in LC3 (38%) had favorable outcomes compared to other luminal-papillary tumors (*p* = 0.003; Fig. 1c, d), whereas stratifying the basal-squamous subtype by lncRNA clusters did not reveal differences in outcome (*p* = 0.66; Additional file 2: Figure S3). Given the enrichment of luminal-papillary tumors in LC3, we named this group of patients “Luminal-Papillary LncRNA Cluster 3 (LPL-C3),” and the other luminal-papillary tumors as “LPL-Other.”

Next, we repeated the consensus clustering in the TCGA cohort (*n* = 405) using the lncRNAs that were consistent between the array and RNA-seq platforms (739/750). This resulted in a four-cluster consensus solution that was substantially concordant with the published TCGA lncRNA results [12] (*κ* = 0.77, *p* < 0.001, Additional file 1: Table S2). As in the NAC cohort, we identified a distinct lncRNA cluster (LC3) enriched in luminal-papillary tumors (74/88 patients, *p* < 0.001) with favorable prognosis (*p* = 0.022) (Additional file 2: Figure S4a-c and Additional file 1: Table S3).

### The biological characteristics of LPL-C3 tumors are consistent with less-aggressive disease

**Fig. 2:**
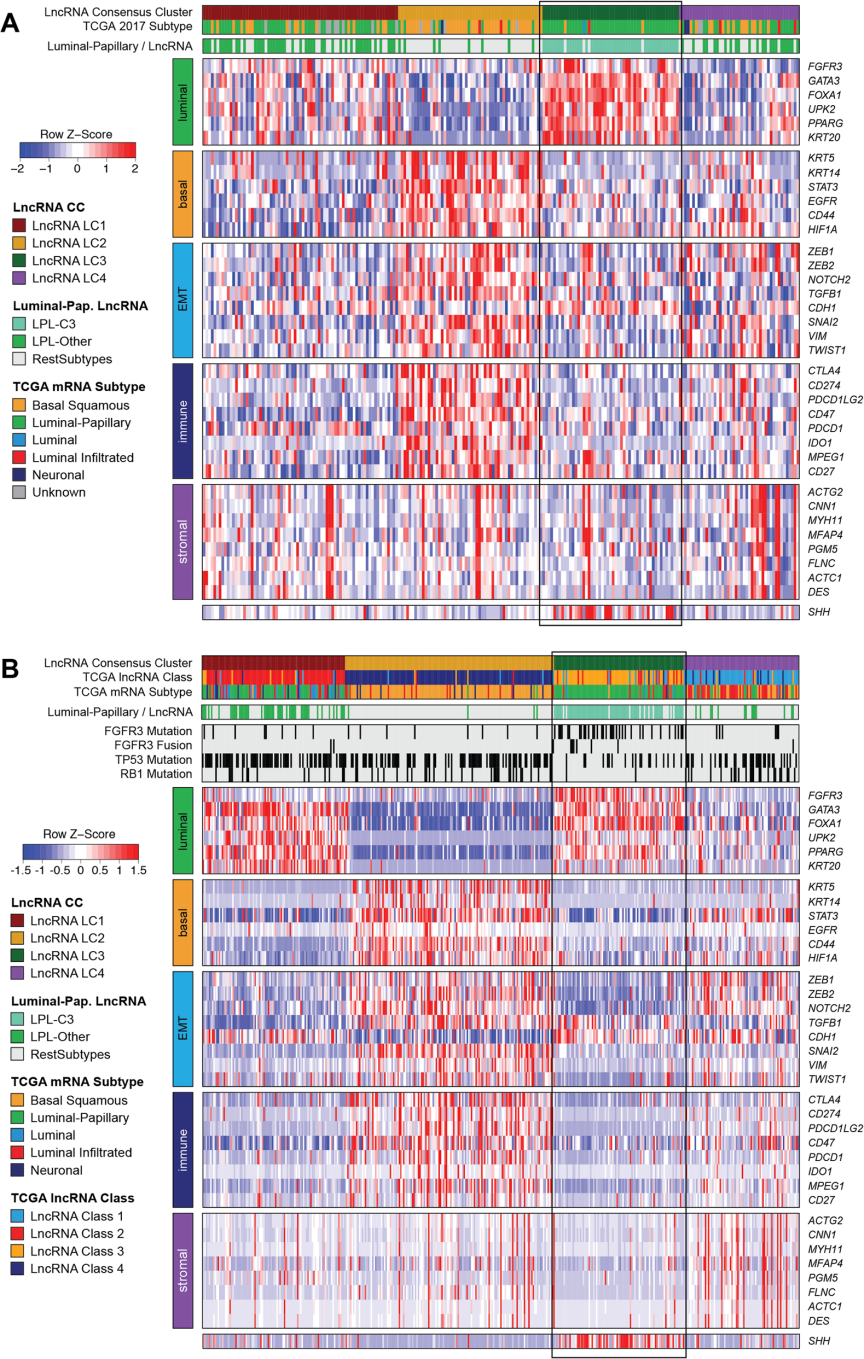
Biological characterization of the lncRNA clusters using selected MIBC marker genes in the **a** NAC and **b** TCGA cohorts. For the NAC and TCGA cohorts, both the five TCGA subtypes (luminal-papillary, luminal, luminal-infiltrated, basal squamous, and neuronal, unknown) and the luminal-papillary subgroups (LPL-C3, LPL-Other and RestSubtypes) are indicated in the covariate tracks. In the TCGA cohort, the 2017 TCGA four-cluster lncRNA solution, *FGFR3*, *TP53*, and *RB1* mutation status and FGFR3 fusion status, are also indicated in covariate tracks

To investigate the biological differences between the LPL-C3 and LPL-Other tumors, we generated a heatmap of genes associated with MIBC subtypes for both the NAC and TCGA cohorts (Fig. 2a, b). Many luminal markers (i.e., *PPARG*, *FOXA1*, and *GATA3*) were expressed at significantly higher levels in LPL-C3 than in LPL-Other tumors (Additional file 2: Figure S5A-C). These patterns were less evident in the TCGA cohort, with only *FOXA1* showing significantly increased expression (*p* = 0.023) (Additional file 2: Figure S5d-f). In both cohorts, all luminal-papillary tumors showed downregulation of basal (i.e., *KRT5/6*, *KRT14*) (Fig. 2a, b and Additional file 2: Figure S6) and immune-associated genes (i.e., *CD274*, *PDCD1LG2*) (Fig. 2a, b and Additional file 2: Figure S7).

**Fig. 3:**
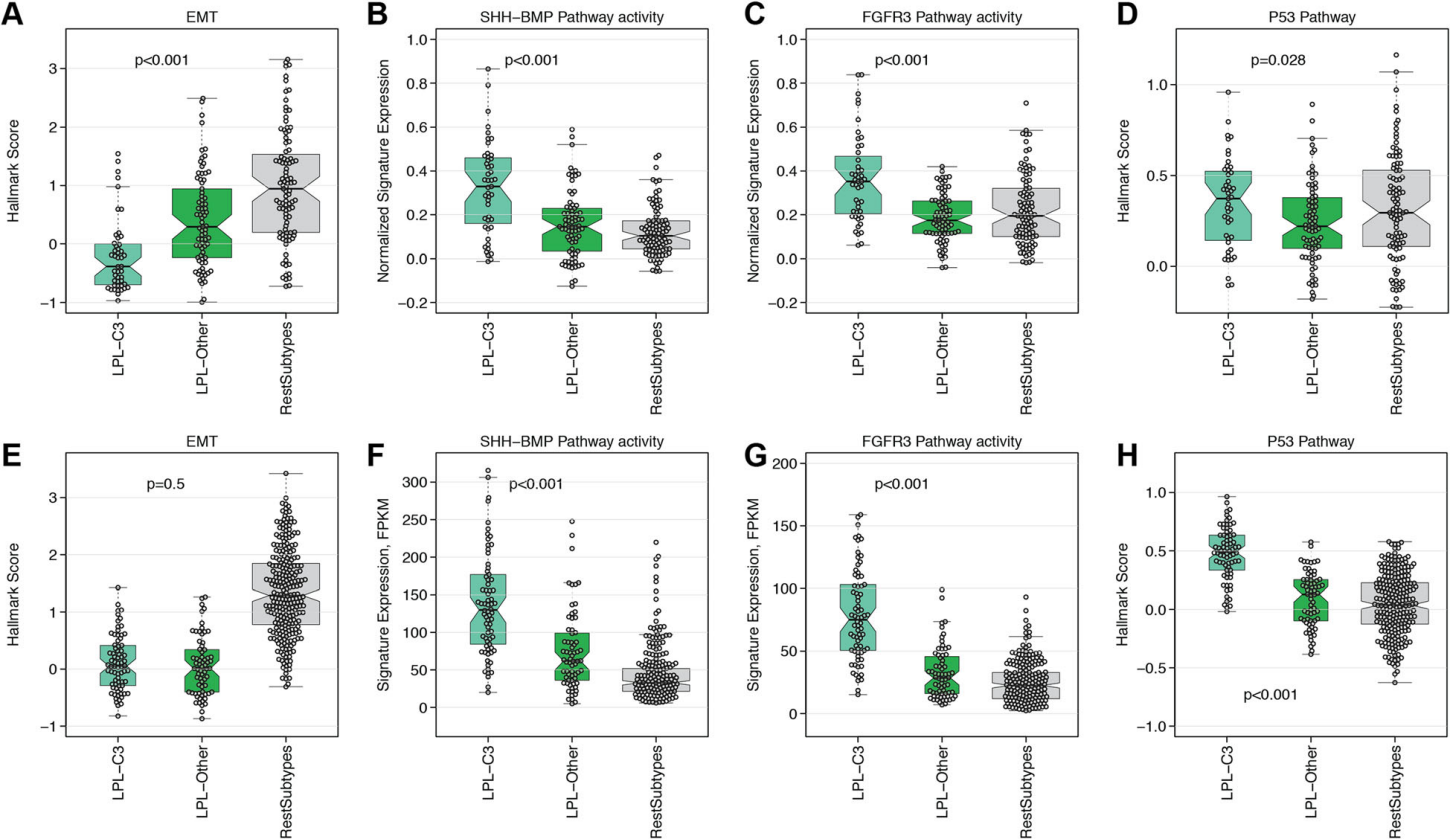
Biological pathways differentially regulated between LPL-C3 and LPL-Other tumors. For the NAC cohort, **a** EMT hallmark activity, **b** SHH-BMP pathway activity, **c** FGFR3 signature score, and **d** p53 hallmark activity. The TCGA cohort follows the same order for panels **e**-**h**

Significant differences in expression of genes associated with epidermal-to-mesenchymal transition (EMT) were observed for LPL-C3 versus LPL-Other tumors in the NAC cohort (Additional file 2: Figure S8a-c). For example, *VIM* and *ZEB1* were less abundant and *CDH1* was more abundant in LPL-C3, indicating lower EMT activity in these tumors. Hallmark EMT signature scores were also significantly lower among the LPL-C3 tumors in the NAC cohort (Fig. 3a). However, in the TCGA cohort, EMT activity differences between LPL-C3 and LPL-Other tumors were not significant (*p* = 0.5), although both luminal-papillary subsets showed low levels of both EMT gene expression and EMT hallmark scores (Fig. 3e and Additional file 2: Figure S8d-f). Moreover, we found LPL-C3 tumors had the highest median purity in both cohorts (Additional file 2: Figure S9), suggesting a general lack of fibroblast infiltration, which may account for the low EMT scores (Additional file 2: Figure S10). As differential immune cell infiltration may have contributed to the lncRNA profiles, we generated a list of immune-enriched lncRNAs and compared these to the 750 initially used for clustering. Only 23 were leukocyte-associated and selected for clustering, although their expression was not limited to the immune-enriched CC2 (Additional file 2: Figure S11).

Higher expression of *SHH* and genes associated with urothelial differentiation (i.e., *UPK3A*, *UPK3B*) are features of luminal-papillary tumors [12, 22]. In both cohorts, LPL-C3 tumors had higher expression of *SHH* (Additional file 2: Figure S12) and SHH-BMP pathway activity signature scores (Fig. 3b, f).

**Fig. 4:**
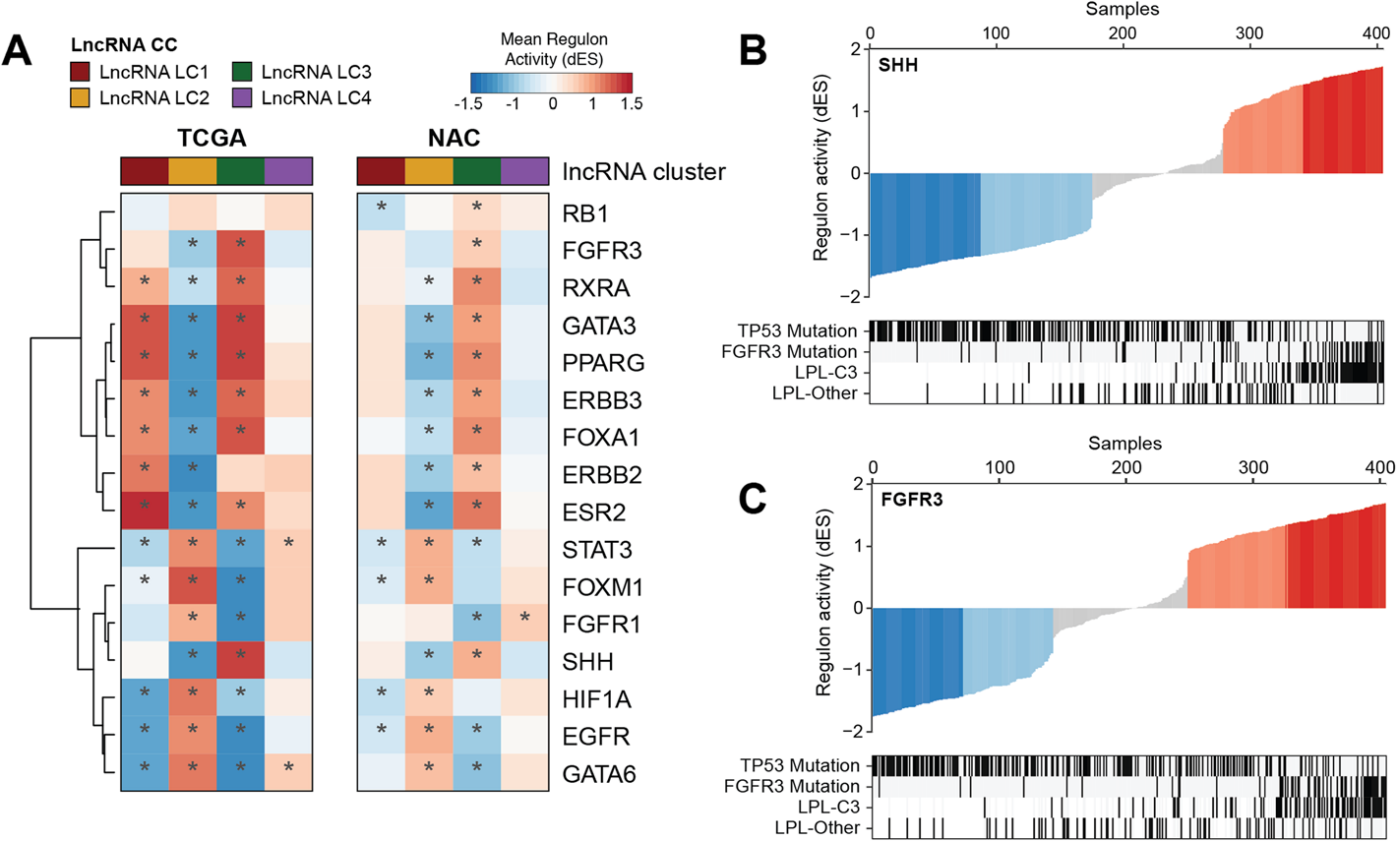
Regulon activities of the lncRNA-based consensus clusters. **a** Mean regulon activities in lncRNA clusters for 16 regulators in the TCGA and NAC cohorts. Asterisks mark clusters that were significantly enriched (Fisher’s exact test, Benjamin Hochberg adjusted, *p* < 10^−3^) with activated or repressed samples for a regulon. Regulons activities in the TCGA cohort for **b** SHH and **c** FGFR3, with *TP53*, *FGFR3*, and *RB1* mutation status and LPL-C3 vs. LPL-Other indicated in covariate tracks. A dark black bar indicates a mutation event

Next, we sought to use regulon activities to further explore the differences in biology between the LPL-C3 tumors, the LPL-Other tumors, and the rest of the cohort [12, 20], using the TCGA cohort for discovery and the NAC cohort for validation. Regulon analysis returns a profile of the activity of a transcription factor (or similar regulator) across a cohort (Methods). Mean regulon activities for LC2 and LC3 subtypes were largely consistent between cohorts, though only weakly for LC1 (Fig. 4a). Activated SHH and FGFR3 regulon activity was associated with LC3 (LPL-C3) tumors and enriched with FGFR3 mutations (Fig. 4b, c), consistent with the results of the gene expression analysis.

### LPL-C3 tumors are enriched for FGFR3 alterations and have wild-type p53 activity

We assessed a panel of 59 genes with mutation status reported in the TCGA cohort [12]. After adjusting for false discovery rate (FDR), we retained *FGFR3*, *TP53*, and *RB1*, whose rates of mutation differed (*p* < 0.05) between LPL-C3 and the rest of the cohort (Fig. 2b and Additional file 1: Table S4).

In the LPL-C3 tumors, the enrichment for *FGFR3*-mutations (33/74 cases, *p* < 0.001) correlated with both increased *FGFR3* gene expression and signaling activity (Additional file 2: Figure S13a, b). These tumors were also enriched for *FGFR3* fusions (6/74, *p* = 0.02; Fig. 2b), which was the only significant fusion event identified when comparing LPL-C3 and the rest of the cohort (Additional file 1: Table S5). Tumors with strongly activated FGFR3 regulon activity were likewise enriched in *FGFR3* mutations, supporting this observation (Fig. 4c). Although *FGFR3* mutation status was not available for the NAC cohort, both the *FGFR3* gene expression and gene signature activity were significantly higher in the LPL-C3 tumors (*p* < 0.001) (Fig. 3c).

To examine if *TP53* mutation correlated with impaired p53 activity, we first compared expression of the p53 pathway hallmark scores between *TP53* mutated and wild-type patients within the TCGA cohort (Additional file 2: Figure S13c, d). The LPL-C3 tumors, which were depleted for *TP53* mutations, showed the highest p53 hallmark scores, which suggested functional p53 activity (Fig. 2b and Fig. 3h). Consistent with this, samples with high SHH and FGFR3 regulon activities were depleted in *TP53* mutation (Fig. 4b, c). Unfortunately, the TP53 regulon had insufficient (< 15) positive and negative targets and was therefore too small to support activity calculations. The TP53 regulon was therefore excluded from the analysis. Although *TP53* mutation status was not available for the NAC cohort, the LPL-C3 tumors had higher p53 hallmark scores, suggesting these tumors may also be depleted for *TP53* mutations (Fig. 3g).

Although LPL-C3 tumors from the TCGA cohort were depleted for *RB1* mutations, *RB1* gene expression differed only non-significantly between LPL subgroups (*p* = 0.054) (Fig. 2b and Additional file 2: Figure S14a). In contrast, LPL-C3 tumors from the NAC cohort had significantly higher expression of *RB1* (*p* = 5.5 × 10^− 4^) (Fig. 2a and Additional file 2: Figure S14b). In contrast to SHH and FGFR3 regulon activities, tumors with higher RB1 regulon activity showed only weak depletion for *TP53* mutations in the TCGA cohort (Additional file 2: Figure S14c).

All genes and pathway activities of LPL-C3 tumors suggested that these tumors should be less clinically aggressive. Therefore, we compared the clinical features of luminal-papillary patients in the NAC cohort and found higher rates of organ-confined disease, including significantly lower pT-stage (*p* = 0.047) and fewer lymph node metastases (*p* = 0.0016) for LPL-C3 tumors (Table 2). Notably, LPL-C3 patients with clinical node involvement still had a good prognosis (Additional file 2: Figure S15). Similar observations were seen in the TCGA cohort, with lower ypT-stage (*p* = 0.0043) and fewer lymph node metastases in LPL-C3 patients (*p* = 0.002). In the NAC and TCGA cohorts, the median age of patients with LPL-C3 tumors was significantly lower (median age 58 vs. 63 years and 61 vs. 70 years, respectively; *p* < 0.01).

**Table 2:** Clinicopathological characteristics of luminal-papillary MIBC patients from the NAC and TCGA cohorts

Luminal-papillary subset	NAC		*p* value	TCGA		*p* value
	LPL-C3 (*n* = 47)	LPL-Other (*n* = 77)		LPL-C3 (*n* = 74)	LPL-Other (*n* = 68)	
Age, median [IQR]	58 (51–65)	63 (58–72)	0.00098	61 (54–71)	70 (64–77)	0.0034
Gender (%)						
Female	12 (26%)	19 (25%)		16 (22%)	14 (21%)	
Male	35 (74%)	58 (75%)	1.00	58 (78%)	54 (79%)	1.00
Clinical lymph node stage (%)						
cN0	36 (77%)	35 (45%)		*NA*	*NA*	
cN1–3	11 (23%)	42 (55%)		*NA*	*NA*	
cNx	0 (0%)	0 (0%)	0.00075	*NA*	*NA*	*NA*
Clinical tumor stage (%)						
Tis/Ta/T1						
cT2	23 (49%)	33 (43%)		*NA*	*NA*	
cT3	18 (38%)	29 (38%)		*NA*	*NA*	
cT4	6 (13%)	15 (19%)	0.64	*NA*	*NA*	*NA*
Pathological tumor stage (%)						
ypT0/Tis/Ta/T1	28 (59%)	32 (42%)		0	0	
ypT2	13 (28%)	17 (22%)		44 (59%)	21 (31%)	
ypT3	5 (11%)	20 (26%)		16 (22%)	28 (41%)	
ypT4	1 (2%)	7 (9%)		7 (9.5%)	6 (9%)	
ypTx	0 (0%)	1 (1%)	0.047	7 (9.5%)	13 (19%)	0.0043
Pathological lymph node stage (%)						
yN0	39 (83%)	35 (45%)		61 (82%)	36 (53%)	
yN1–3	5 (11%)	24 (31%)		6 (8%)	17 (25%)	
yNx	3 (6%)	18 (23%)	0.0016	7 (9%)	15 (22%)	0.0020

### Development of a single-sample classifier to identify luminal-papillary MIBC patients with good prognosis

To provide utility as a prognostic model, we developed a single-sample genomic classifier (GC) to identify the good-prognosis luminal tumors with activated FGFR3 (FGFR3+). To be classified as FGFR3+, the tumor must also show enhanced SHH activity, higher p53 pathway activity, and lower EMT, consistent with the data shown above.

**Fig. 5:**
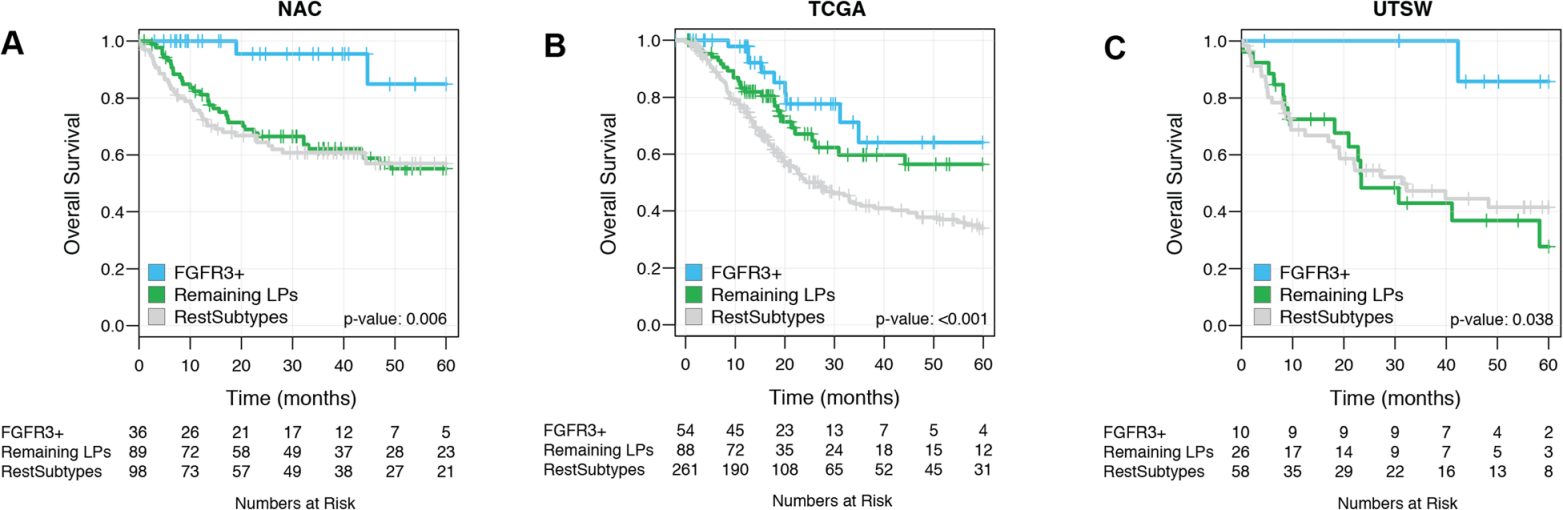
Survival analysis of FGFR3+ cases determined by the GC in three cohorts. **a** NAC (*n* = 223), **b** TCGA (*n* = 405), and **c** UTSW (*n* = 94)

We identified 36/223 (16%) and 55/408 (14%) FGFR3+ cases in the NAC and TCGA cohorts, respectively. The majority of the FGFR3+ calls in both cohorts were of the luminal-papillary mRNA subtype (Additional file 1: Table S6). In both cohorts, patients with FGFR3+ tumors had better survival than other patients (*p* = 0.001 and *p* = 0.003 for NAC and TCGA, respectively) (Fig. 5a, b). As expected, we found the FGFR3, SHH, and p53 signature scores were significantly higher among FGFR3+ cases when comparing them to the other tumors. In the NAC cohort, EMT hallmark scores were significantly lower among FGFR3+ cases (*p* < 0.001), whereas FGFR3+ cases from the TCGA cohort showed no significant difference in EMT activity (Additional file 2: Figure S16A-H). *FGFR3* was mutated in 25/55 FGFR3+ cases (45%) compared to 32/350 negative cases (9%) from the TCGA cohort (*p* < 0.001). The FGFR3+ cases were depleted for TP53 mutations in 15/55 (27%) compared to 180/350 (51%) negative cases (*p* < 0.001). Likewise, RB1 mutations were fewer in FGFR3+ cases, 0/55 (0%) compared to 70/350 (20%) of negative cases (*p* < 0.001).

To validate the classifier, we used an independent RC cohort (UTSW) of 94 patients, identifying 10 (11%) FGFR3+ cases (all luminal-papillary) with excellent prognosis (Fig. 5c) and expected biological character (Additional file 2: Figure S17a-d). Multivariable Cox regression analysis revealed that the GC was a significant survival predictor in the NAC TURBT cohort, but not in the TCGA and UTSW cohorts (Additional file 1: Table S7). The GC was also validated in a prospectively collected commercial cohort (PCC, *n* = 225), resulting in 24/225 (11%) FGFR3+ cases (21 luminal-papillary, 3 luminal) with genomic characteristics consistent with FGFR3+ cases from the other cohorts (Additional file 2: Figure S17f-i). Unfortunately, follow-up data were unavailable for this cohort and therefore outcomes could not be determined.

### Comparison of the GC single-sample classifier to the consensus subtyping model

Finally, we also used the recently released consensus molecular classification of The Bladder Cancer Molecular Taxonomy Group to assign tumors from all four cohorts into the six consensus mRNA subtypes (Ba/Sq, LumNS, LumP, LumU, Stroma-rich, and NE-like). Intersecting the consensus subtype calls with the results of the GC revealed that our GC identified tumors from all three luminal subtypes (unstable, non-specified, or papillary), and only rarely the stromal-rich consensus subtype (Additional file 1: Table S8).

## Discussion

Molecular characterization of MIBC by transcriptome profiling has revealed a range of subtypes with distinct clinicopathological characteristics, prognosis, and response to therapeutic regimens. Significant efforts have been invested in mRNA-based molecular subtyping of MIBC; however, mRNA transcripts represent only 1–2% of the transcriptome, which is dominated by ribosomal RNA and ncRNAs [26]. In non-muscle-invasive bladder cancer (NMIBC), lncRNA and mRNA expression appear to correlate with each other [27], although only TCGA has explored stratification of MIBC using the non-coding transcriptome [12].

In the present study, we selected a list of highly variable lncRNAs for consensus clustering and identified a subset of luminal-papillary MIBC patients with favorable prognosis (LPL-C3). This lncRNA-mediated subdivision of the luminal-papillary mRNA subtype was consistent with, though not identical to, the TCGA lncRNA clustering solution [12]. LncRNA expression has been described as highly specific to tissue, cell, or disease state, compared to mRNAs [28, 29]; these data support the utility of lncRNA expression in refining mRNA-based subtyping models. Although we observed differential immune infiltration in our lncRNA clusters, only a handful of lncRNAs highly expressed in lymphocytes were identified in our lncRNA set used for clustering, suggesting these were not major contributors to the signal driving the clustering solution.

As the current work was an independent analysis using a panel of *de novo* selected lncRNAs, these data demonstrate that the lncRNA transcriptome contains additional signal for the identification of a biologically distinct MIBC subgroup with potential clinical utility. This highlights a significant advancement over mRNA-based subtyping, where the additional granularity in the subtypes resulted in meaningful survival associations. Notably, LPL-C3 patients with clinically node-positive disease, who would be expected to have *worse* outcomes, also were found to have surprisingly good outcomes. Thus, the identification of a group of patients with superior prognosis is a major finding that significantly advances the bladder cancer field.

The LPL-C3 tumors had genomic features consistent with less-aggressive disease, including wild-type p53 activity, FGFR3 activation, and lower EMT. LncRNAs have been implicated in the p53-regulatory network in colorectal, nasopharyngeal, and prostate cancers [30–32], where they function as regulators [33, 34]. Some of the lncRNAs that we used in our unsupervised clustering may reflect a wild-type p53 network, facilitating the identification of the LPL-C3 subgroup. Effective cell cycle/apoptosis regulation by p53 may confer a less-aggressive tumor and the favorable prognosis seen in patients with these tumors.

In bladder cancer, *TP53* and *FGFR3* mutations are reported to be mutually exclusive [35, 36]. In the TCGA cohort, tumors in the LPL-C3 group, while being depleted for *TP53* mutations, had *FGFR3* mutation rates five times higher than in other tumors. These tumors also showed higher levels of *FGFR3* gene expression, pathway activation, and regulon activity, consistent with the mutational activation of FGFR3 [37]. Mutations in *FGFR3* have been reported in bladder cancer to be associated with a less-aggressive disease, lower-stage tumors, and improved prognosis, consistent with the data from our study [36, 38].

Other biological features may also explain the less-aggressive clinical course of patients with LPL-C3 tumors. In these tumors, we observed higher expression of *SHH* and downstream SHH targets, and higher expression of the *SHH* gene has been proposed to restrain bladder cancer progression [22, 39]. Moreover, in the NAC cohort, the LPL-C3 tumors had lower EMT activity, a feature known to be associated with less-aggressive cancer in many tumor types [40]. In the TCGA cohort, both LPL-C3 and LPL-Other tumors had lower EMT activity, suggesting this feature may be a characteristic of the luminal-papillary subtype.

Collectively, the luminal nature of the LPL-C3 tumors, the wild-type p53 activity, the high proportion of *FGFR3* mutations, SHH-BMP pathway activity, and lower EMT signature all support a less-aggressive tumor type and suggest a biological explanation for the favorable prognosis of patients with these tumors. However, the extent of the LPL-C3/FGFR3+ survival benefit differed between the NAC and TCGA cohorts, which may be caused by a different treatment regimen (NAC+RC versus RC only), as the survival curves of all four lncRNA clusters were shifted upwards in the NAC cohort. In contrast, FGFR3+ patients from the UTSW (RC only) cohort showed even better prognosis than FGFR3+ cases from the NAC cohort, despite having had a different treatment regimen. Additionally, over half of the tumors in the TCGA cohort are pT3/T4, which may explain, at least in part, the less favorable outcomes seen for these patients.

While MIBC has a poor prognosis in general, identifying a subgroup of patients with excellent outcomes would be a major step in addressing the heterogenous clinical behavior of this disease. In daily clinical practice, such patients could be offered a less invasive treatment. To provide clinical utility for our findings, we developed a stringent, single-sample classifier that identified FGFR3+ cases with high FGFR3 activity and enrichment for *FGFR3* mutations/fusions. Early results from a phase II trial showed a 40% overall response rate in patients with FGFR3-mutated, metastatic urothelial cancer after treatment with erdafitinib, an FGFR inhibitor [41]. Consequently, FGFR3+ cases may be candidates for treatment with FGFR3 inhibitors instead of NAC, as patients with luminal tumors may benefit less from NAC while still being exposed to chemotherapy-related toxicity [11].

This retrospective study has several limitations. First, DNA sequence data was unavailable for the NAC, UTSW, and PCC cohorts, so we were unable to accurately determine whether the LPL-C3 (or FGFR3+) cases were enriched for *FGFR3* mutations or depleted for *TP53* mutations. Although the FGFR3 signature is a reasonable surrogate, and FGFR3 regulon activities show promise as a complementary metric, availability of mutation calls for patients from all cohorts would strengthen the study. Second, the PCC cohort lacked clinical follow-up, so we were only able to evaluate the GC model calls based on genomics.

In the TCGA and UTSW cohorts, the HR, although not statistically below the *p* value threshold of 0.05, was consistently below 0.50 in all datasets tested, suggesting a protective status for FGFR3+ tumors. For UTSW, the cohort was small (*n* = 94) with only 10 FGFR3+ patients, which may explain why FGFR3+ status did not achieve significance in multivariable analysis. Given the reported trends, we anticipate that statistical significance may be achieved with additional patients. For the TCGA cohort, sufficient tumor tissue for the many different assays required by TCGA studies (copy number, RNA-seq, DNA methylation, etc.) may have resulted in the collection of larger, more bulky tumors which tend to exhibit a more aggressive clinical behavior. For our study, the FGFR3+ tumors may therefore be on the more aggressive side of the spectrum of the LPL-C3 tumors, resulting in a higher HR than observed in the NAC or UTSW cohort, and possibly explaining the lack of a significant *p* value in the TCGA survival analysis.

Given these factors, the GC will require additional prospective validation before it can be used clinically as a single-sample classifier for identifying luminal-papillary MIBC patients with enhanced FGFR3 activity and favorable prognosis.

## Conclusions

In summary, using the lncRNA transcriptome, we identified a subgroup of luminal-papillary MIBC patients that have very good outcomes. We characterized these tumors genomically, and biologically, and characterized the patients clinically. Further, we developed a single-sample genomic classifier to identify such tumors and validated it in two independent cohorts.

## Additional files

Additional file 1: **Table S1**: Final list of gene features and coefficients for the genomic classifier. **Table S2**: Concordance of our generated lncRNA clusters and the lncRNA clusters from the TCGA 2017 publication, in the TCGA cohort. **Table S3**: Intersection of the lncRNA-based consensus clusters and the TCGA2017 mRNA subtypes for the NAC and TCGA cohorts. **Table S4**: Mutation status enrichment for LPL-C3 vs other in TCGA cohort. **Table S5**: Fusion event enrichment for LPL-C3 vs other in TCGA cohort. **Table S6**: Intersection of the GC FGFR3+ tumors and the TCGA2017 mRNA subtypes for the NAC, TCGA, UTSW and PCC cohorts. **Table S7**: Multivariable analyses in the NAC, TCGA and UTSW cohorts. **Table S8**: Intersection of the GC FGFR3+ tumors and the Consensus Classifier mRNA subtypes for the NAC, TCGA, UTSW and PCC cohorts. (XLSX 23 kb)

Additional file 2: **Figure S1**: Consensus cluster plus output from unsupervised clustering of 750 highly variant lncRNAs in the NAC cohort. **Figure S2**: Molecular subtyping of the NAC cohort using the TCGA 2017 classifier. **Figure S3**: Survival analysis for lncRNA clusters stratified by the basal/squamous mRNA subtype in the NAC cohort. **Figure S4**. Survival analysis for lncRNA clusters in TCGA cohort. **Figure S5**: Expression of select MIBC marker genes associated with the luminal subtype in the LPL-C3 and LPL-Other tumors. **Figure S6**: Expression of select MIBC marker genes associated with the basal subtype in the LPL-C3 and LPL-Other tumors. **Figure S7**: Expression of select MIBC marker genes associated with the immune oncology in the LPL-C3 and LPL-Other tumors. **Figure S8**: Expression of select genes associated with EMT in the LPL-C3 and LPL-Other tumors. **Figure S9**: Sample purity estimates. **Figure S10**: Scatterplots for the observed correlation between EMT hallmark scores and purity estimates. **Figure S11**: Contribution of immune-associated lncRNAs to consensus clustering solution in the NAC cohort. **Figure S12**: Expression of select genes associated with SHH and urothelial differentiation in the LPL-C3 and LPL-Other tumors. **Figure S13**: Correlation of gene expression and pathway activity with respect to mutation status in the TCGA cohort. **Figure S14**: RB1 expression. **Figure S15**: Survival analysis of LPL-C3 patients stratified by node positivity. **Figure S16**: Biological pathways differentially activated between tumors classed as FGFR3+ by the GC and other tumors. **Figure S17**: Biological pathways differentially active between tumors classed as FGFR3+ by the GC and other tumors. (PDF 4062 kb)

## Data Availability

The datasets analyzed during the current study are available in the Gene Expression Omnibus repository (http://www.ncbi.nlm.nih.gov/geo/), under accession codes GSE87304 and GSE128702 [11, 17]. The TCGA RNA-seq data was available as part of the TCGA research network (http://cancergenome.nih.gov/) [12]. The PCC cohort is available for non-commercial research through a data usage agreement with the test manufacturer [17]. Requests can be made via email (partner@decipherbio.com). The lymphocyte and normal bladder expression datasets are available in the GTEx Portal (https://gtexportal.org/).
