# Supplementary material for: Long non-coding RNAs identify a subset of luminal muscle-invasive bladder cancer patients with favorable prognosis
Source: Genome Med. 2019 Oct 17;11:60. doi: 10.1186/s13073-019-0669-z (PMC6796434; doi:10.1186/s13073-019-0669-z)
Supplement: Supplementary file 2 — Figure S1: Consensus cluster plus output from unsupervised clustering of 750 highly variant lncRNAs in the NAC cohort. Figure S2: Molecular subtyping of the NAC cohort using the TCGA 2017 classifier. Figure S3: Survival analysis for lncRNA clusters stratified by the basal/squamous mRNA subtype in the NAC cohort. Figure S4. Survival analysis for lncRNA clusters in TCGA cohort. Figure S5: Expression of select MIBC marker genes associated with the luminal subtype in the LPL-C3 and LPL-Other tumors. Figure S6: Expression of select MIBC marker genes associated with the basal subtype in the LPL-C3 and LPL-Other tumors. Figure S7: Expression of select MIBC marker genes associated with the immune oncology in the LPL-C3 and LPL-Other tumors. Figure S8: Expression of select genes associated with EMT in the LPL-C3 and LPL-Other tumors. Figure S9: Sample purity estimates. Figure S10: Scatterplots for the observed correlation between EMT hallmark scores and purity estimates. Figure S11: Contribution of immune-associated lncRNAs to consensus clustering solution in the NAC cohort. Figure S12: Expression of select genes associated with SHH and urothelial differentiation in the LPL-C3 and LPL-Other tumors. Figure S13: Correlation of gene expression and pathway activity with respect to mutation status in the TCGA cohort. Figure S14: RB1 expression. Figure S15: Survival analysis of LPL-C3 patients stratified by node positivity. Figure S16: Biological pathways differentially activated between tumors classed as FGFR3+ by the GC and other tumors. Figure S17: Biological pathways differentially active between tumors classed as FGFR3+ by the GC and other tumors. (PDF 4062 kb) [file 13073_2019_669_MOESM2_ESM.pdf]

Additional file 2: Supplementary figures

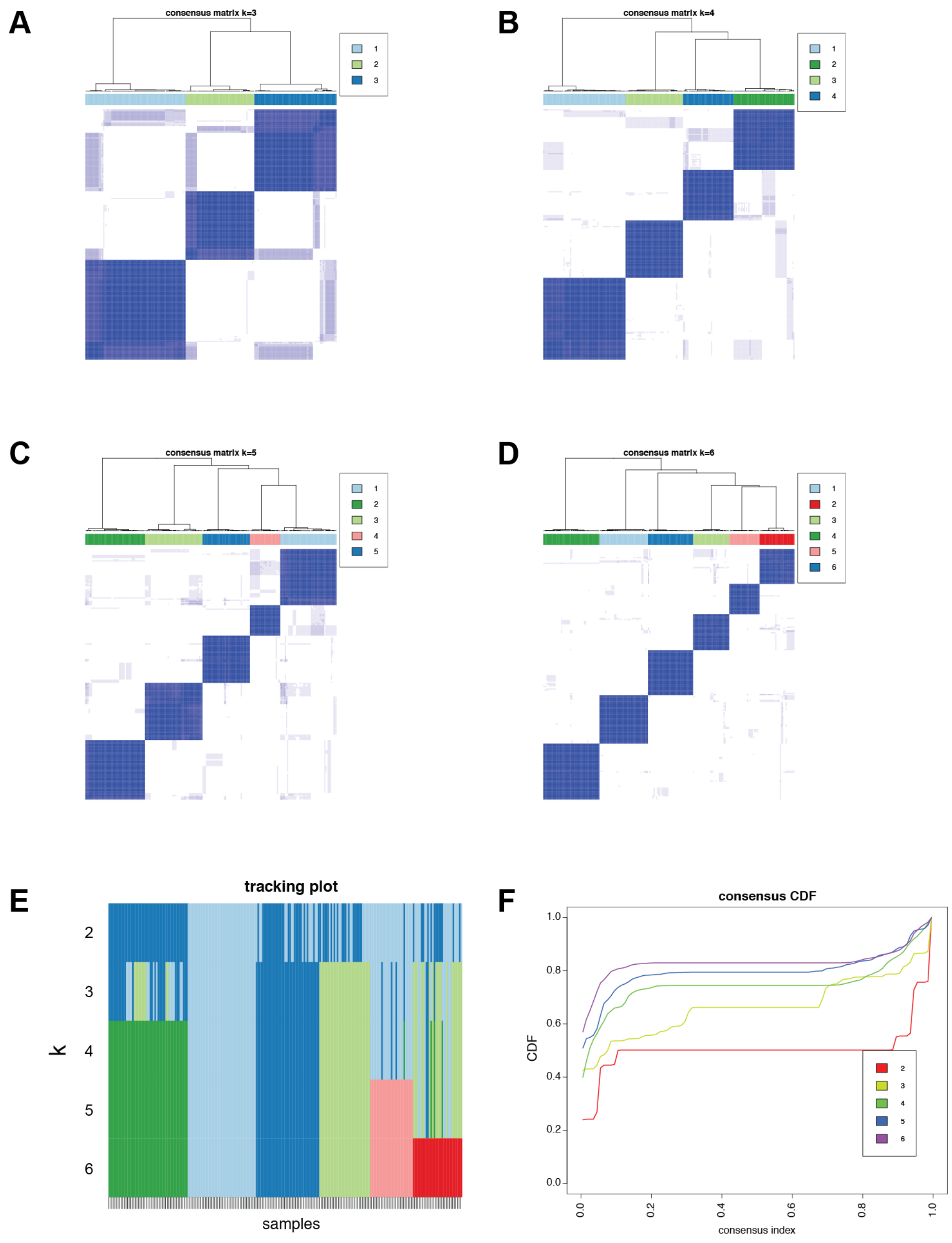

Figure S1: Consensus cluster plus output from unsupervised clustering of 750 highly variant lncRNAs in the NAC cohort. (A-D) Consensus membership heatmaps for the (A) 3-cluster solution, (B) 4-cluster solution, (C) 5-cluster solution, (D) 6-cluster solution, (E) tracking plot, and (F) Cumulative Distribution Function (CDF) plot of consensus membership values for solutions with between 2 and 6 clusters.

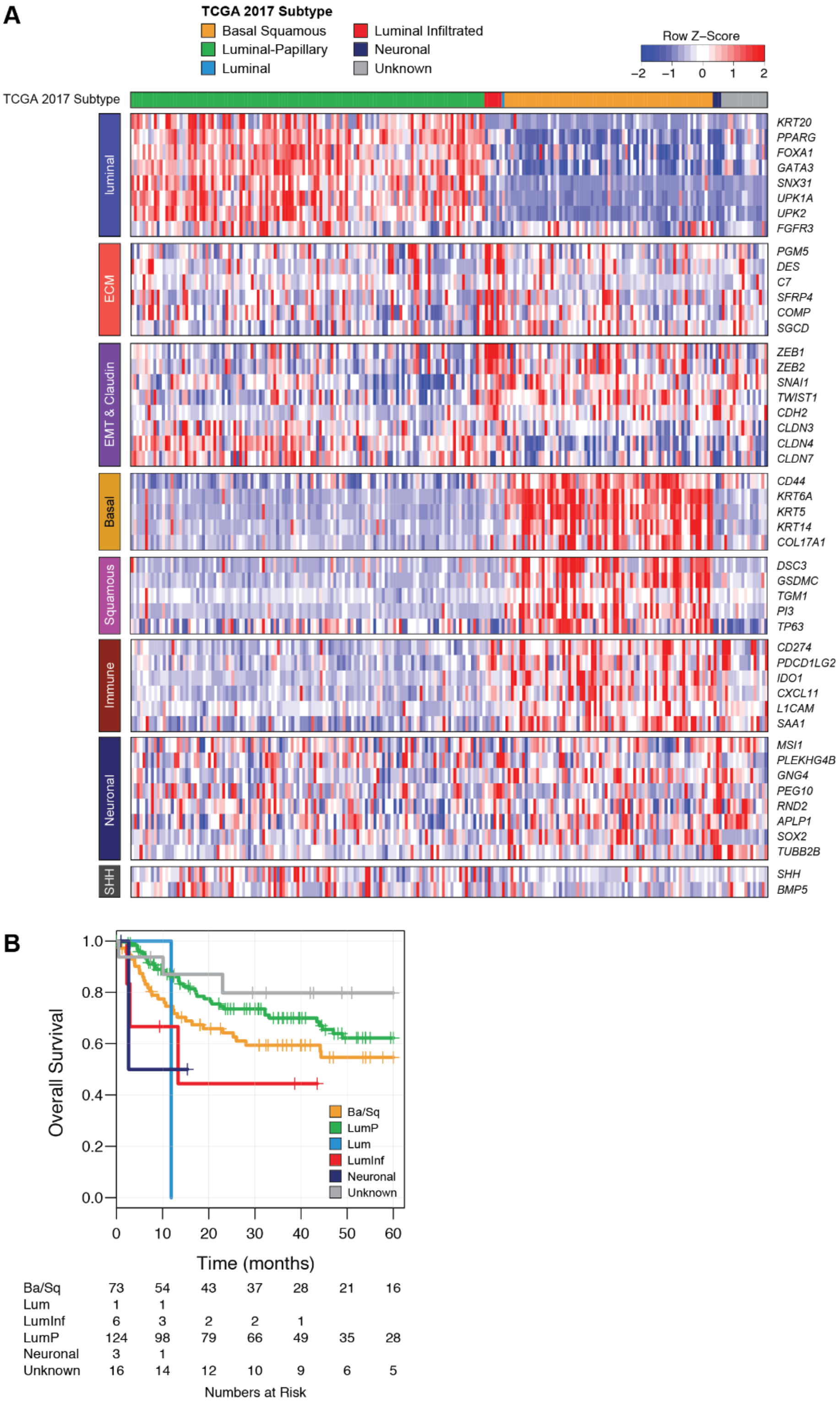

Figure S2: Molecular subtyping of the NAC cohort using the TCGA 2017 classifier. (A) Heatmap of the five TCGA subtypes (luminal-papillary, luminal, luminal infiltrated, basal squamous and neuronal) and unknown. (B) KM plot of the NAC cohort stratified by the TCGA 2017 classifier.

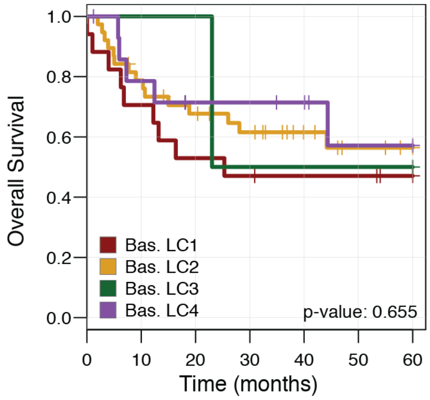

Bas. LC1	17	12	9	8	7	7	4
Bas. LC2	39	29	24	20	13	9	7
Bas. LC3	2	2	2	1	1	1	1
Bas. LC4	15	11	5	8	7	4	4

Numbers at Risk

Figure S3: Survival analysis for lncRNA clusters stratified by the basal/squamous mRNA subtype in the NAC cohort.

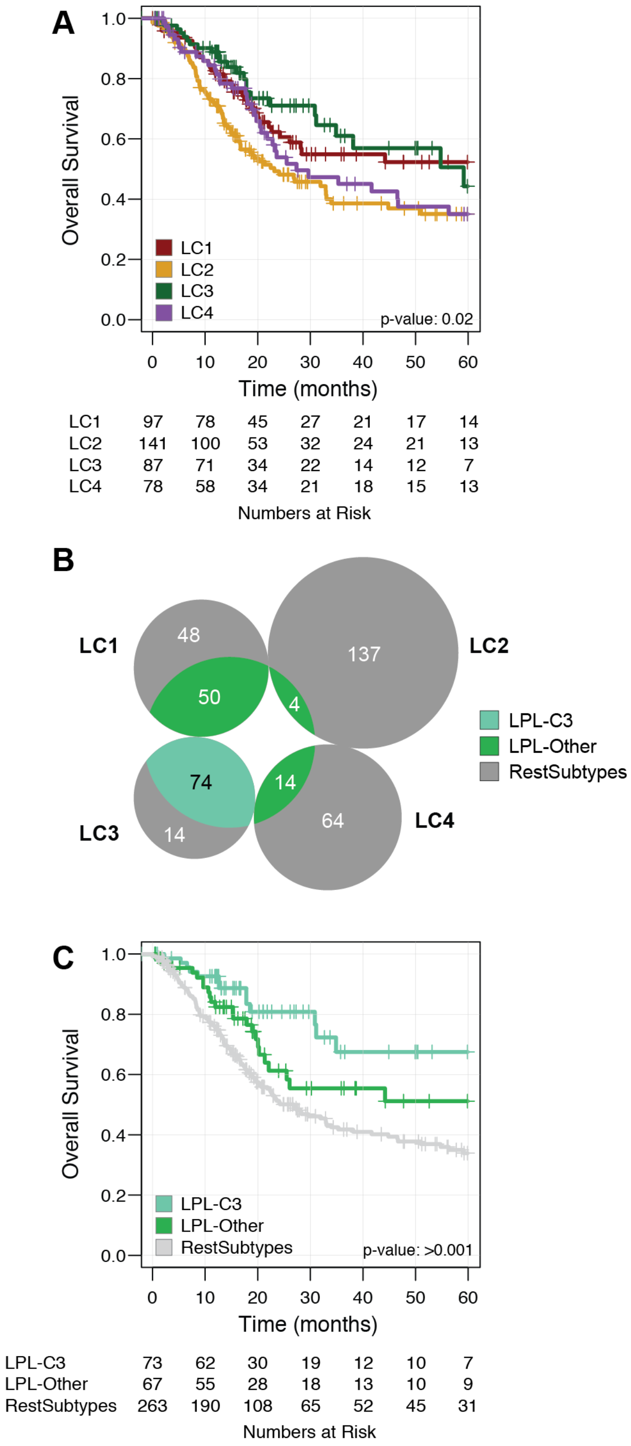

Figure S4. Survival analysis for lncRNA clusters in TCGA cohort. (A) KM plot for lncRNA clusters (LC1-4) in the TCGA cohort, (B) Intersection of the lncRNA clusters (LC1-4) with the luminal papillary mRNA subtype and (C) KM plot for lncRNA-split luminal papillary tumors (LPL-C3, LPL-Other).

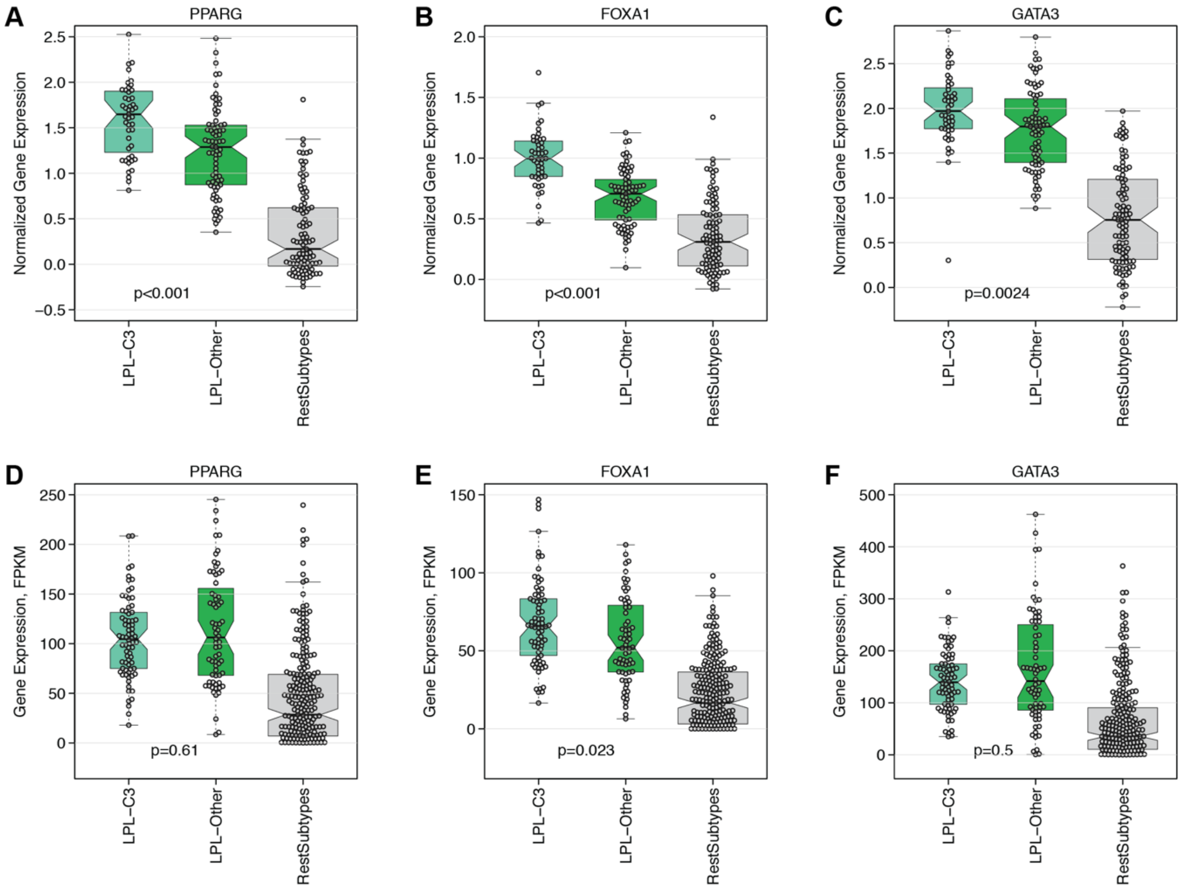

Figure S5: Expression of select MIBC marker genes associated with the luminal subtype in the LPL-C3 and LPL-Other tumors, for the NAC cohort (A) *PPARG*, (B) *FOXA1*, (C) *GATA3* and TCGA cohort (D) *PPARG*, (E) *FOXA1*, (F) *GATA3*, respectively.

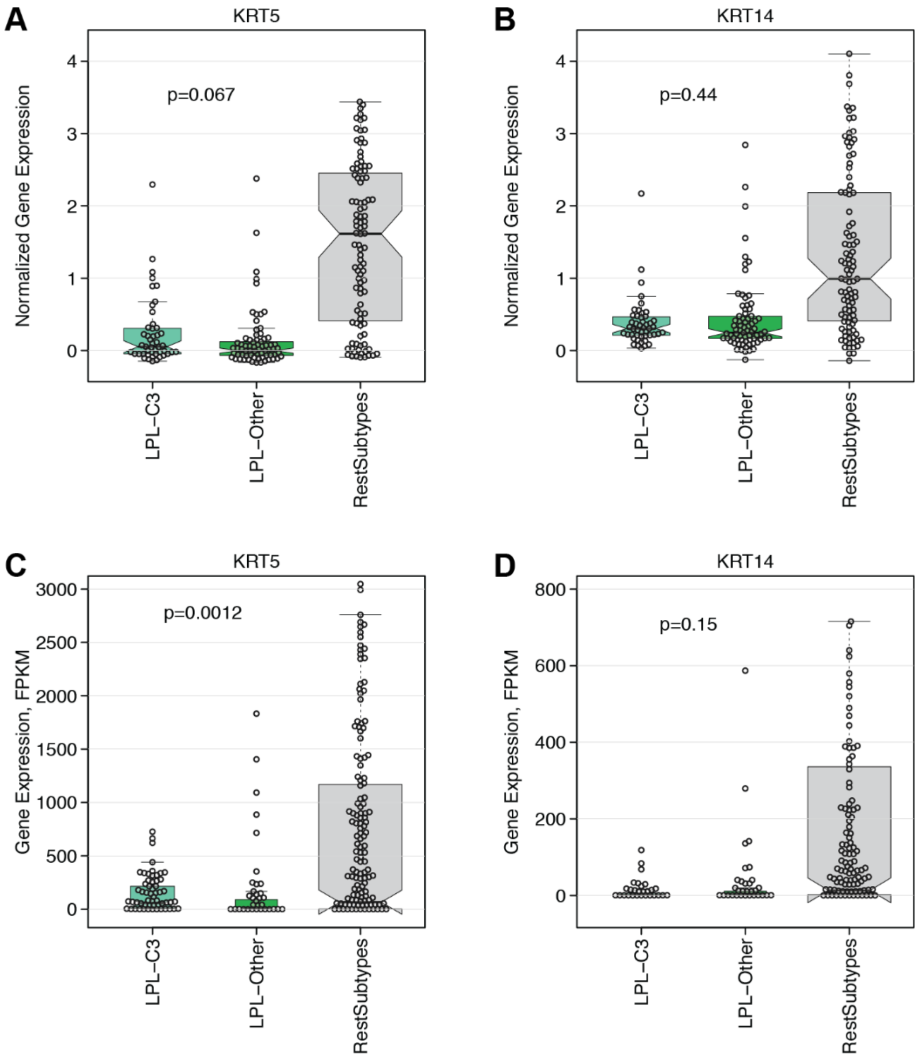

Figure S6: Expression of select MIBC marker genes associated with the basal subtype in the LPL-C3 and LPL-Other tumors, for the NAC cohort (A) *KRT5*, (B) *KRT14* and TCGA cohort (C) *KRT5*, (D) *KRT14*, respectively.

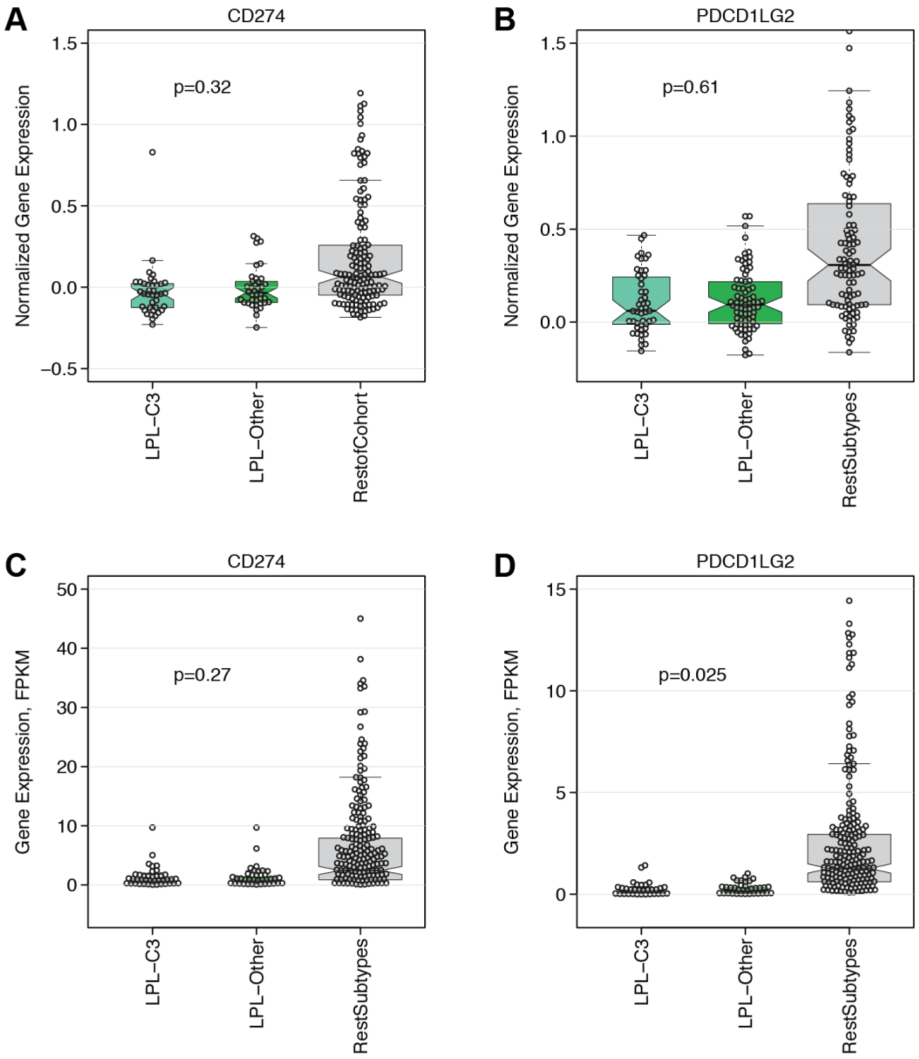

Figure S7: Expression of select MIBC marker genes associated with the immune oncology in the LPL-C3 and LPL-Other tumors, for the NAC cohort (A) *CD274* (PD-L1), (B) *PDCD1LG2* (PD-1) and TCGA cohort (C) *CD274* (PD-L1), (D) *PDCD1LG2* (PD-1), respectively.

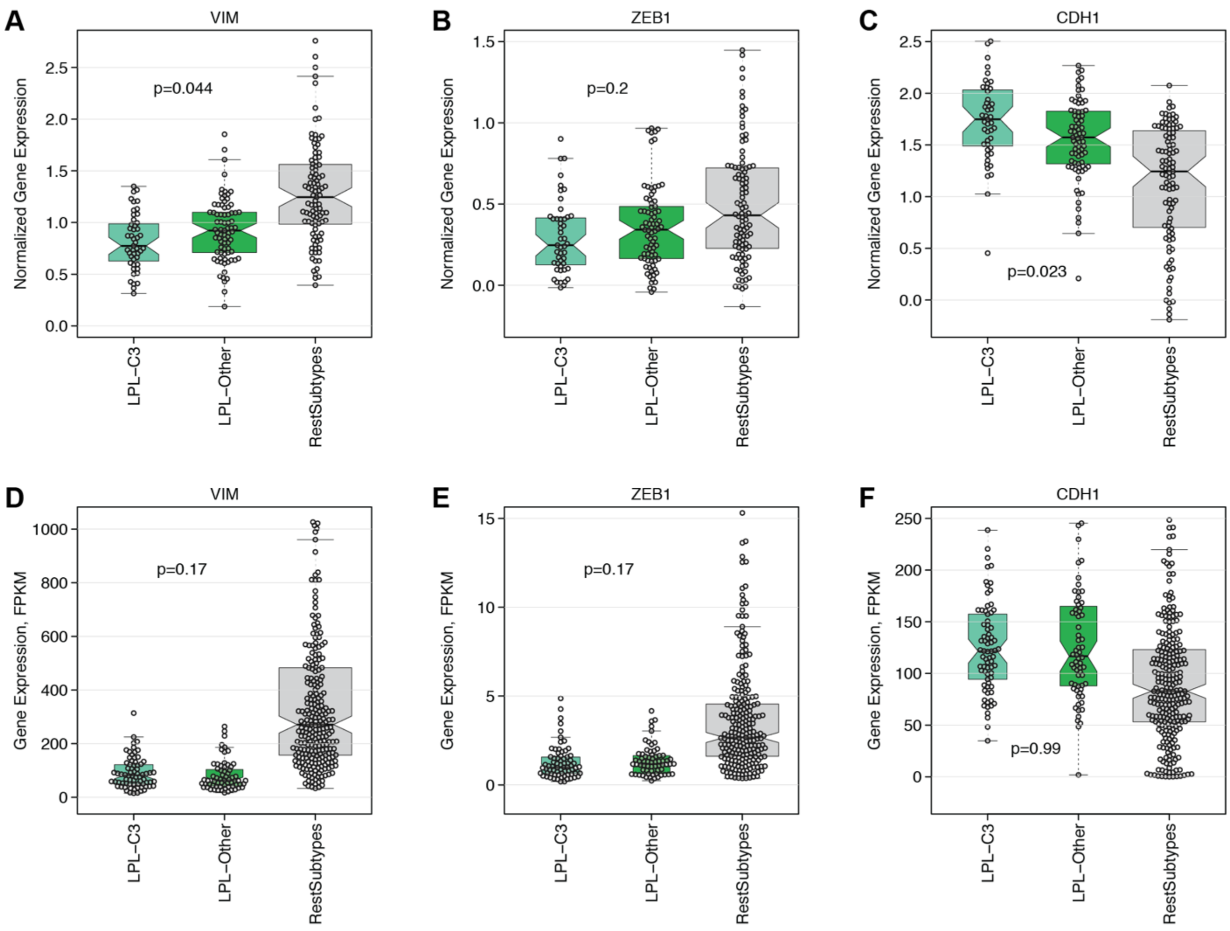

Figure S8: Expression of select genes associated with EMT in the LPL-C3 and LPL-Other tumors, for the NAC cohort (A) *VIM*, (B) *ZEB1*, (C) *CDH1* and TCGA cohort (D) *VIM*, (E) *ZEB1*, (F) *CDH1*, respectively.

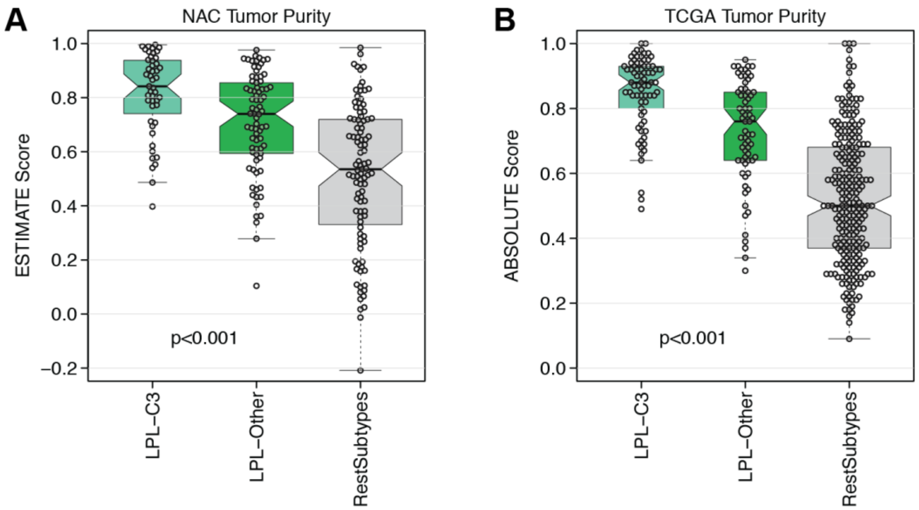

Figure S9: Sample purity estimates for the (A) NAC cohort using the ESTIMATE algorithm and (B) TCGA using the ABSOLUTE algorithm (previously calculated).

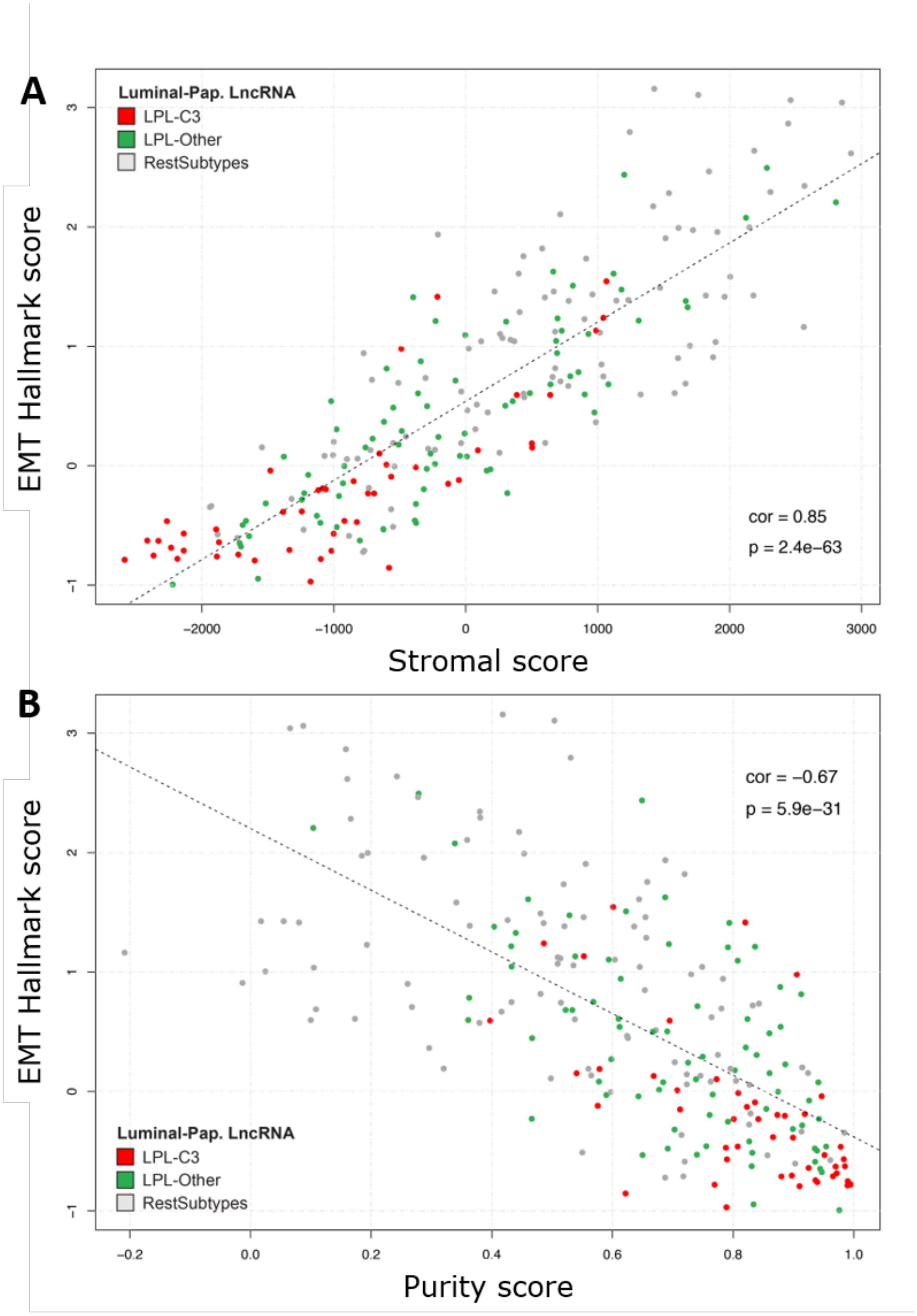

Figure S10: Scatterplots for the observed correlation between EMT hallmark scores and (A) stromal and (B) purity estimates, using the NAC cohort and the ESTIMATE algorithm.

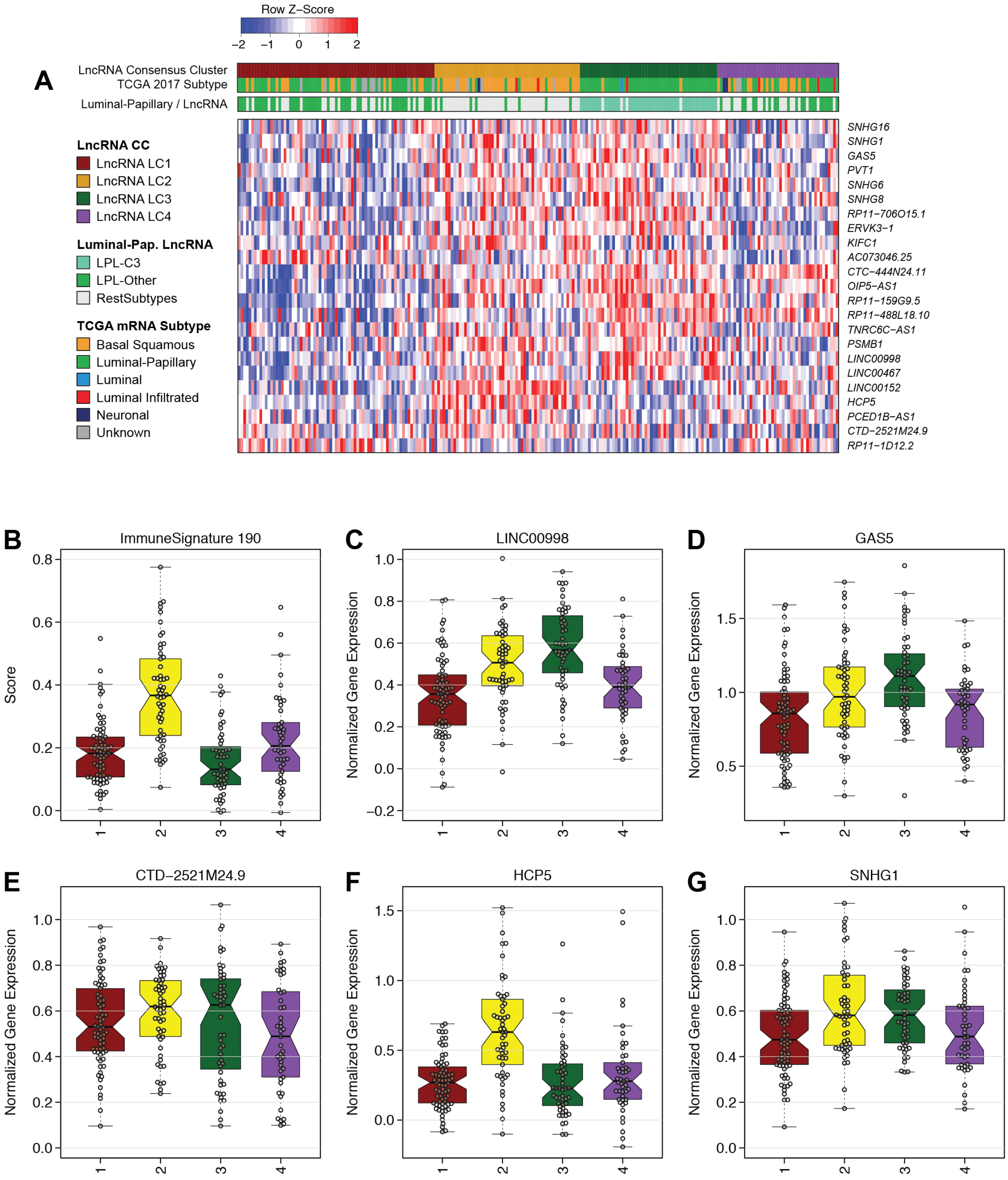

Figure S11: Contribution of immune-associated lncRNAs to consensus clustering solution in the NAC cohort. (A) Heatmap of 23 lncRNAs highly expressed in lymphocytes and contributing to lncRNA-clustering. (B) Immune190 signature score indicating immune-infiltration levels. LncRNA expression levels for (C) *LINC00998*, (D) *GAS5*, (E) *CTD-2521M24.9*, (F) *HCP5* and (G) *SNGH1*.

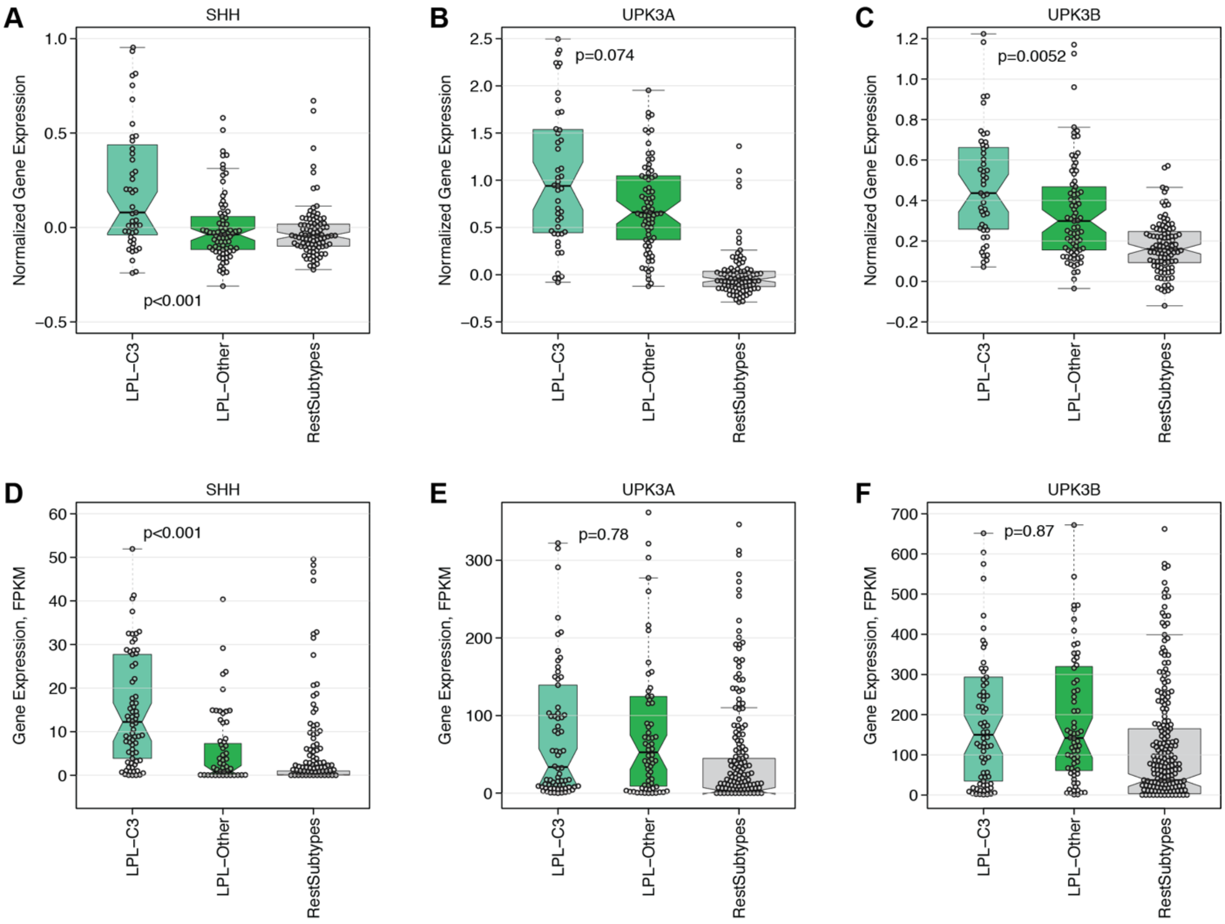

Figure S12: Expression of select genes associated with SHH and urothelial differentiation in the LPL-C3 and LPL-Other tumors, for the NAC cohort (A) *SHH*, (B) *UPK3A*, (C) *UPK3B* and TCGA cohort (D) *SHH*, (E) *UPK3A*, (F) *UPK3B*, respectively.

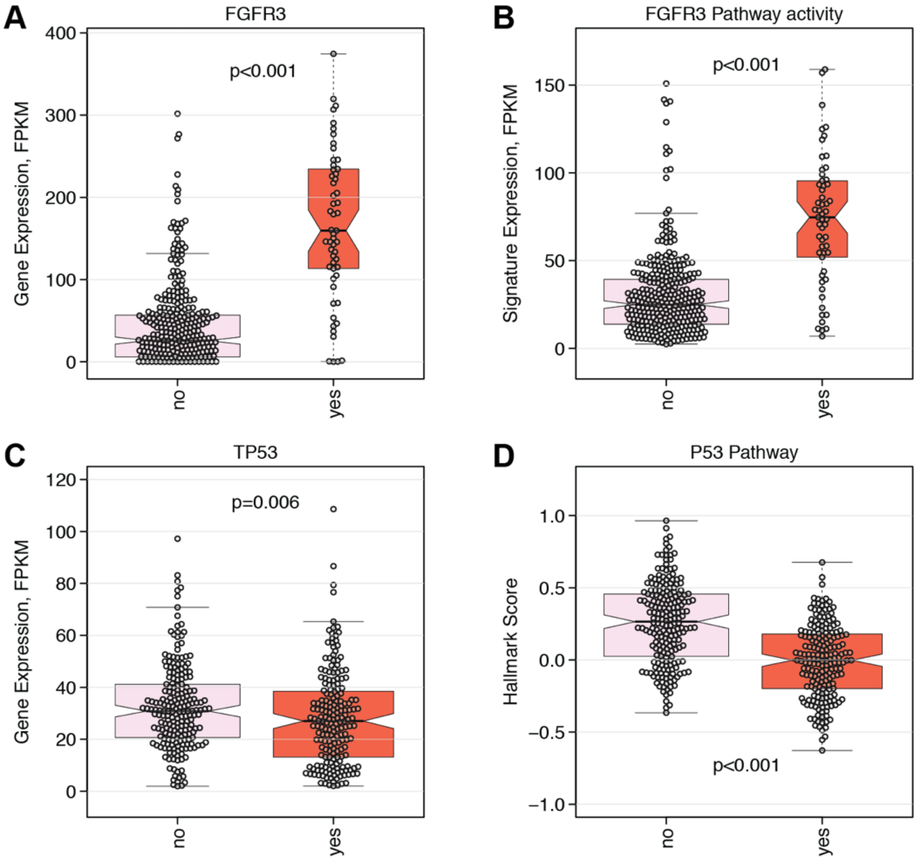

Figure S13: Correlation of gene expression and pathway activity with respect to mutation status in the TCGA cohort. (A) *FGFR3* gene expression, (B) FGFR3 pathway activity, (C) *TP53* gene expression and (D) p53 pathway activity. For each panel, ‘no’ indicates wild-type and ‘yes’ indicates a mutation.

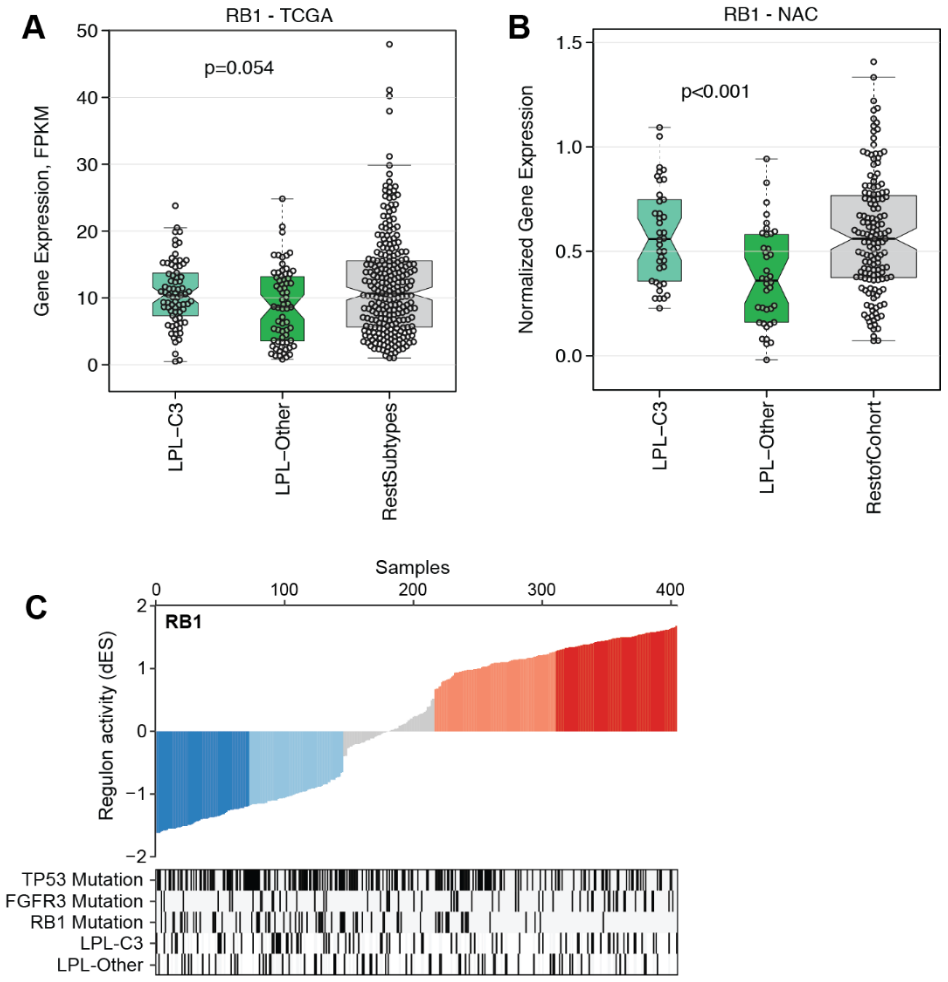

Figure S14: RB1 expression in (A) TCGA and (B) NAC cohorts. (C) RB1 regulon activity scores in TCGA cohort with *TP53*, *FGFR3* and *RB1* mutation status and LPL-C3 vs. LPL-Other indicated in covariate tracks. A dark black bar indicates a mutation event.

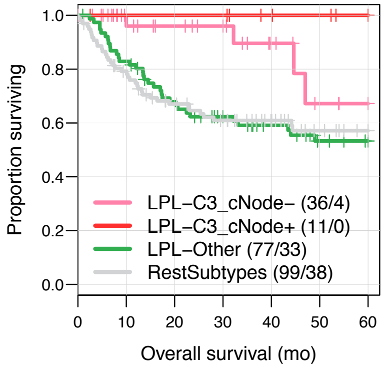

Figure S15: Survival analysis of LPL-C3 patients with (LPL-C3 cNode+) and without (LPL-C3 cNode-) clinically node positive disease.

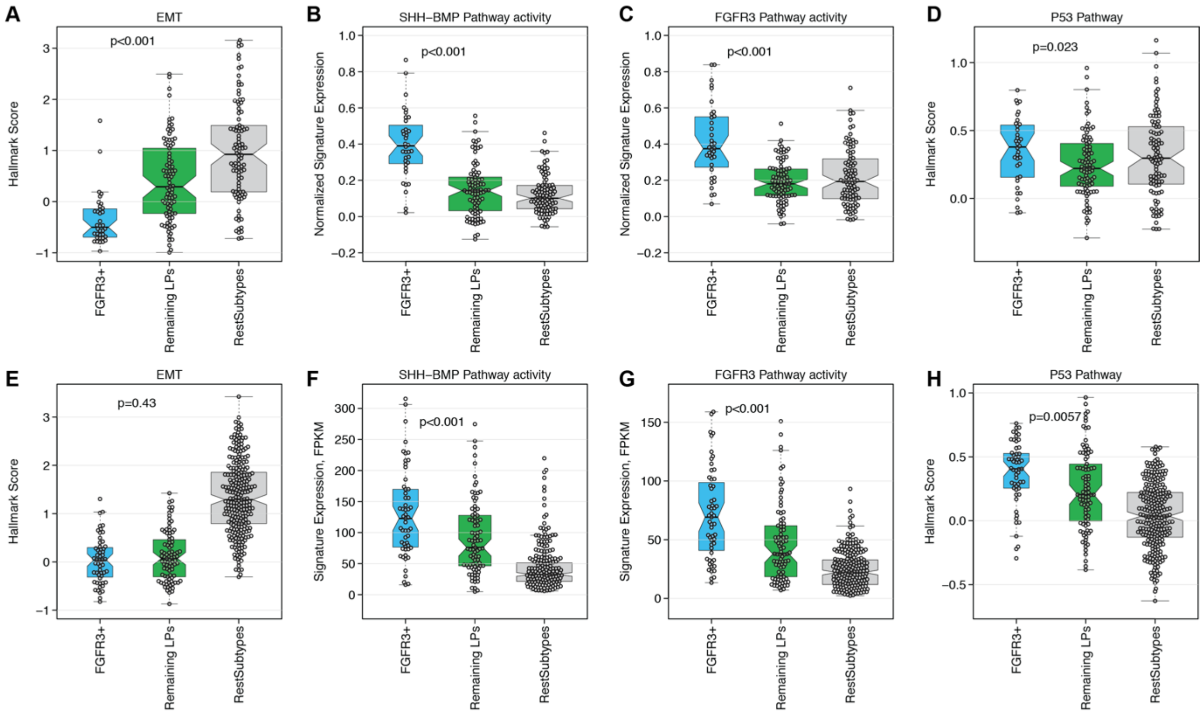

Figure S16: Biological pathways differentially activated between tumors classed as FGFR3+ by the GC and other tumors. For the NAC cohort (A) EMT hallmark activity, (B) SHH-BMP pathway activity, (C) FGFR3 signature score, (D) p53 hallmark activity. The TCGA cohort follows the same order for panels E-H.

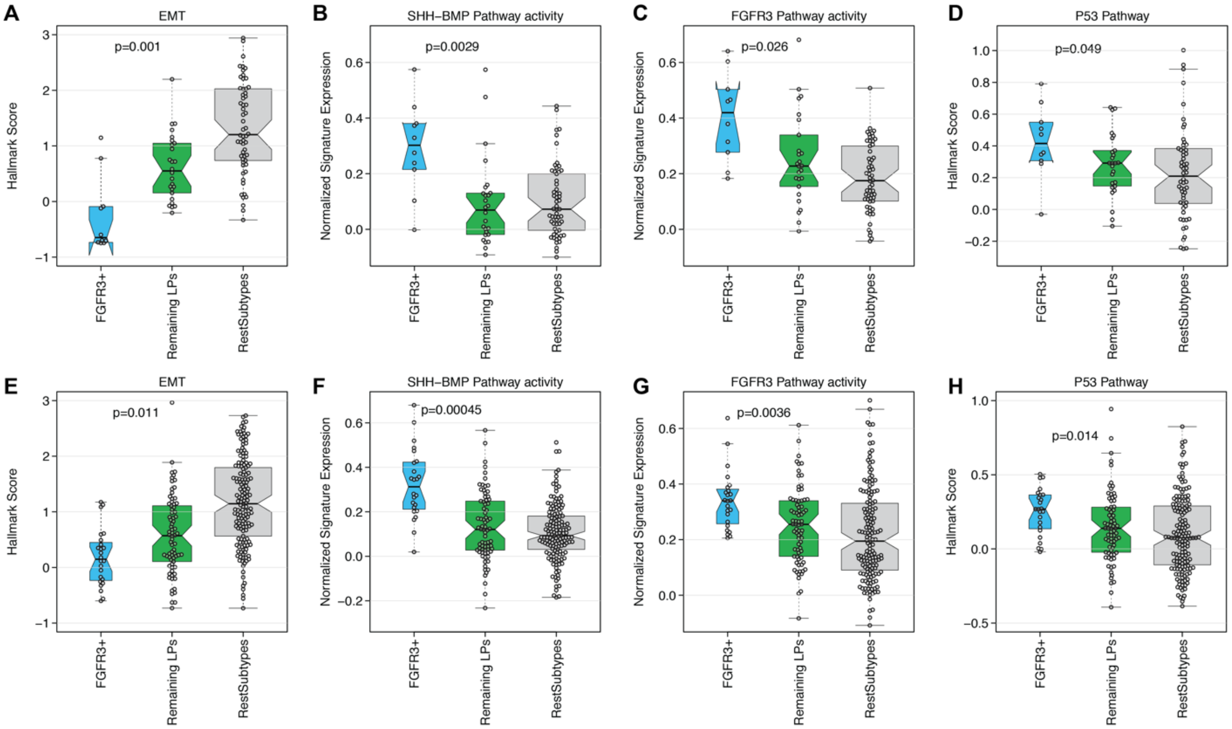

Figure S17: Biological pathways differentially active between tumors classed as FGFR3+ by the GC and other tumors. For the UTSW cohort (A) EMT hallmark activity, (B) SHH-BMP pathway activity, (C) FGFR3 signature score, (D) p53 hallmark activity. The PCC cohort follows the same order for panels E-H.
